# Modifying Microenvironment in Van der Waals Gap by Cu/N Co‐Doping Strategy for Highly Efficient Nitrite Reduction to Ammonia

**DOI:** 10.1002/advs.202417773

**Published:** 2025-02-24

**Authors:** Heen Li, Yuanzhe Wang, Kuo Wei, Maoyue He, Mengmeng Yan, Fei Peng, Faming Gao

**Affiliations:** ^1^ Tianjin Key Laboratory of Multiplexed Identification for Port Hazardous Chemicals Tianjin University of Science & Technology Tianjin 300222 P. R. China; ^2^ Key Laboratory of Applied Chemistry Yanshan University Qinhuangdao 066004 P. R. China; ^3^ Analyses and Testing Center Hebei Normal University of Science and Technology Qinhuangdao 066000 P. R. China

**Keywords:** 2D material, electrocatalyst, nitrite reduction, SnS_2_

## Abstract

Electroreduction of nitrite to ammonia has significant promise for economical NH_3_ electrosynthesis and wastewater treatment. Herein, sulfur vacancies rich Cu─N co‐doped SnS_2_ nanosheet is designed as a highly active and durable NO_2_RR catalyst. Benefiting from the Cu─N co‐doped strategy, Cu/N‐SnS_2‐x_ achieves the highest NH_3_ yield rate of 18.15mg h^−1^ mg_cat_^−1^ at −0.935 V (vs RHE) and excellent Faradaic Efficiency of 95.73% at −0.835 V (vs RHE). In situ FT‐IR and in situ XPS proves that Cu/N‐SnS_2‐x_ has a greater capacity for atomic hydrogen generation, which facilitates the conversion of nitrite to ammonia and maintains excellent structural stability during the NO_2_RR process. Theoretical calculations reveal that the introduced sulfur vacancies effectively expose the metal atoms inside SnS_2_ and make them adsorb nitrite efficiently, which effectively accelerates the transformation of nitrite to ammonia. Besides, the introduced Cu and N can form a new electronic structure, which induces Cu in an electron‐deficient state promotes the adsorption of reaction intermediates on Cu, and reduces the reaction energy barrier for nitrite reduction on the Cu/N‐SnS_2‐x_ surface. The current exploration presents fresh prospects for the rational development of an effective electrocatalyst for synthesizing ammonia from nitrite.

## Graphical abstract

In this work, the co‐introduction of Cu and N efficiently improves the NO_2_RR performance of pristine‐SnS_2_. Cu, N co‐doped SnS_2_ with abundant sulfur vacancies which effectively enhance the conversion of nitrite and achieves a superior NH_3_ yield of 18.15mg h^−1^ mg_cat_^−1^ and Faradic efficiency of 95.73%.

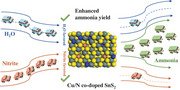

## 1 Introduction

Ammonia (NH_3_), one of the most commonly used industrial products, is in high demand in agriculture and industry due to its fundamental attribute of carbon‐free hydrogen carriers, high‐energy density, and low emissions.^[^ ^1^ ^]^ Currently, NH_3_ is produced industrially by Haber–Bosch process at hightemperatures and pressure, which not only consumes a lot of energy but also produces a lot of CO_2_.^[^ ^2^ ^]^ Electrochemical nitrogen reduction reaction (NRR) is regarded as a green ammonia manufacturing pathway due to its mild reaction conditions and environmental benefits.^[^ ^3^ ^]^ Nevertheless, the insufficient N_2_ conversion as well as the strong nonpolar N_2_ bond and restricted solubility under ambient conditions limit the development of the NRR process.^[^ ^4^ ^]^ Thus, it is critical to discover another promising nitrogen‐containing source to manufacture ammonia, which will play an important role in the future energy system.^[^ ^5^ ^]^ By contrast, electrochemical NH_3_ synthesis via NO_x_^−^ reduction reaction (NO_x_^−^RR) with lower N═O bond energy (204 kJ mol^−1^), which can be obtained from the atmosphere and sewage, is more conducive to efficient NH_3_ synthesis.^[^ ^6^ ^]^ Furthermore, NO_x_^−^ is widespread in groundwater, posing a persistent threat to human health.^[^ ^7^ ^]^ Thus, NO_x_^−^ is a suitable N‐source for ammonia electro‐synthesis from an environmental standpoint.^[^ ^8^ ^]^ However, a major obstacle to the conversion of NO_x_^−^ to NH_3_ is the intricate multi‐electron transfer.^[^ ^9^ ^]^ Therefore, it is particularly desirable to use high‐performance electrocatalysts for selective NH_3_ generation via NO_x_^−^RR.^[^ ^10^ ^]^

Nowadays, 2D materials like graphene,^[^ ^11^ ^]^ transition metal dichalcogenides (TMDs),^[^ ^12^ ^]^ black phosphorus,^[^ ^13^ ^]^ and few‐layer MXenes,^[^ ^14^ ^]^ have gained significant interest in catalytic, electronics, photonics, and optoelectronics applications due to the specific 2D confinement of electron motion and optical properties.^[^ ^15^ ^]^ In contrast to graphene with near zero bandgap and unstable black phosphorus, 2D transition metal dichalcogenides (TMDs) are semiconductors with excellent stability and wide bandgaps that range from 1 to 2.5 eV., which makes TMDs excellent candidates as electrocatalysts.^[^ ^16^ ^]^ Amidst TMDs, SnS_2_ is a semiconductor of the CdI_2_‐type with layered non noble metal sulfide, exhibiting exceptional electrical activity, a sufficient bandgap, and excellent carrier mobility.^[^ ^17^ ^]^ SnS_2_ exhibits significant stability in oxidation resistance in addition to excellent stability in electrolytes.^[^ ^18^ ^]^ However, the intrinsic activity of pristine SnS_2_ still cannot meet the increasing requirement for highly efficient and reliable electrocatalysts for future energy conversion and storage systems, thus it is urgent to further modify SnS_2_ to build a highly efficient NO_x_^−^RR catalyst.

Doping has been one of the most commonly employed techniques for controlling catalytic activity and selectivity when building heterogeneous catalysts.^[^ ^19^ ^]^ The base material coordination environment and electrical configuration can be dramatically changed by adding foreign elements as dopants, which improves the catalytic properties of the material.^[^ ^20^ ^]^ Recent reports elucidate that modification of SnS_2_ by doping with heteroatoms is a viable and effective means of enhancing NO_x_^−^ reduction capacity of SnS_2_.^[^ ^21^ ^]^ Zhang et al., successfully doped Fe into SnS_2_ to form sulfur vacancies rich Fe‐SnS_2_ by hydrothermal method and suggest that the generation of sulfur vacancies greatly enhanced the number of active sites, and the introduction of Fe further adjusted the electronic structure of the metal sites, which effectively lowered the reaction energy barriers of the intermediates in the reduction process of NO_x_^−^ and facilitated the high speed of NH_3_ production, demonstrating that heteroatom doping is an effective method to modify SnS_2_.^[^ ^22^ ^]^ Besides, typical nonmetallic elements (N, P, and S) can effectively balance the adsorption and desorption to intermediates for the redistribution of atomic charge around dopants.^[^ ^23^ ^]^ Therefore, it is a promising catalyst designing a strategy to further introduce new non‐metallic elements into SnS_2_ to enhance its NO_x_^−^ reduction ability.

In line with these requirements, a simple hydrothermal method was applied to introduce Cu and N into the SnS_2_ structure. Benefiting from the Cu─N co‐doped strategy, Cu/N‐SnS_2‐x_ achieves the highest NH_3_ yield rate of 18.15mg h^−1^ mg_cat_^−1^ at −0.935 V(vs RHE) and Faradaic Efficiency of 95.73% at −0.835 V(vs RHE), ESR experiments and in situ FT‐IR test prove Cu/N‐SnS_2‐x_ has a greater capacity for atomic hydrogen generation, which facilitates the conversion of nitrite to ammonia. Theoretical calculations revealed the introduced N element could induce Cu in an electron‐deficient state, which promotes the adsorption of Cu on nitrogen‐containing intermediates and reduces the reaction energy barrier for nitrite reduction. Besides, in situ Raman test and long‐term *i‐t* test prove the excellent stability of Cu/N‐SnS_2‐x_, suggesting the practicality of industrial applications.

## 2 Results and Discussion

**Figure 1:**
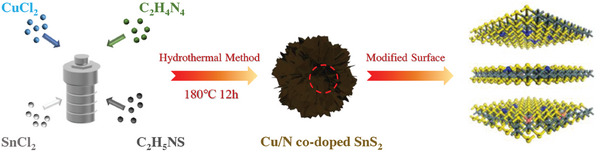
Schematic illustration of the synthesis process of Cu/N‐SnS_2‐x._

Cu/N‐SnS_2‐x_ was synthesized by the one‐step hydrothermal method. The Supporting Information presents additional details about the synthesis procedure (**Figure** **1**).

**Figure 2:**
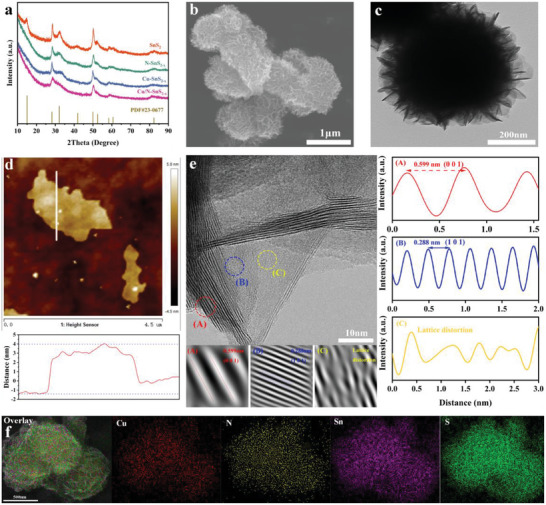
a) XRD patterns. b) SEM image of Cu/N‐SnS_2‐x_. c) TEM image of Cu/N‐SnS_2‐x_. d) AFM image of Cu/N‐SnS_2‐x_. e) HRTEM image of Cu/N‐SnS_2‐x_ and the corresponding line intensity profiles in A, B, and C regions. f) Element mapping images of Cu/N‐SnS_2‐x_.

In order to determine the phase structure and crystallinity of each sample, X‐ray diffraction (XRD) was first applied. As shown in **Figure** **2**a, all sample's peaks have been identified as SnS_2_ (JCPDS No.23‐0677) in the absence of any noticeable contaminants.^[^ ^17a^ ^]^ Furthermore, all the peaks of Cu/N‐SnS_2‐x_, N‐SnS_2‐x_, and Cu‐SnS_2‐x_ are notably weaker compared to those of pristine SnS_2_ indicating the lower crystallinity of Cu/N‐SnS_2‐x_, N‐SnS_2‐x_, and Cu‐SnS_2‐x_ caused by the doping procedure. The morphology of all generated samples was initially investigated using scanning electron microscopy (SEM). SEM images of SnS_2_ (Figure S1a, Supporting Information) and Cu/N‐SnS_2‐x_ (Figure 2b) revealed that synthesized samples were made up of ultrathin nanosheets and were arbitrarily coupled to create nanoflowers and specifically, the large gaps between the nanosheets can provide good electrolyte‐Cu/N‐SnS_2‐x_ interaction, leading to the quick conversion of nitrite to ammonia.^[^ ^24^ ^]^ Transmission electron microscopy (TEM) images further revealed the nanoflower structure of SnS_2_ (Figure S1b, Supporting Information) and Cu/N‐SnS_2‐x_ (Figure 2c) with uniform size.

Atomic force microscopy (AFM) analysis was also performed to estimate the thickness Cu/N‐SnS_2‐x_. As shown in Figure 2d, the topography of the Cu/N‐SnS_2‐x_ reveals a nanosheet morphology with a thickness of ≈4–5 nm which is consistent with the results of SEM and TEM. Besides, the BET result reveals the specific surface area of Cu/N‐SnS_2‐x_ is 320.36 m^2^ g^−1^, suggesting Cu/N‐SnS_2‐x_ exhibits a relatively high surface area (Figure S4, Supporting Information). To further investigate the crystal structure and the existence of sulfur vacancy in Cu/N‐SnS_2‐x_, high‐resolution transmission electron microscopy (HRTEM) was further applied. Figure 2e shows fine lattice fringes with a spacing of 0.599 nm which is assigned to the (001) facet of SnS_2_, suggesting the successful synthesis of SnS_2_, this result is also consistent with the DFT calculation results (Figures S5 and S6, Supporting Information). Line intensity profiles generated from noise‐filtered pictures revealed continuously and evenly ordered lattice atoms in Cu/N‐SnS_2‐x_, indicating the strong crystallinity of Cu/N‐SnS_2‐x_. In addition, the strategy of doping Cu and N significantly reduced the crystallinity of SnS_2_, as evidenced by blurry and discontinuous lattice fringes seen in Cu/N‐SnS_2‐x_ (Selected area C in Figure 2e) and the loss of certain lattice atoms directly supporting the creation of sulfur vacancies.^[^ ^25^ ^]^ Furthermore, as shown in Figure 2f, element mapping images obtained through energy‐dispersive spectroscopy (EDS) show the even contribution of Cu, N, Sn, and S, which strongly confirms the successful introduction of Cu and N.

**Figure 3:**
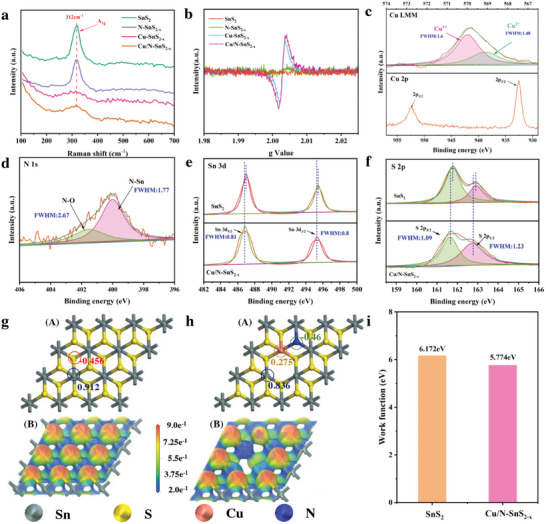
a) Raman patterns. b) ESR patterns. c) XPS Cu2p spectra and Cu LMM AES spectra. d) XPS N 1s spectra. e) XPS Sn3d spectra. f) XPS S2p spectra. g,h) Detailed charge analysis and the corresponding ELF results of SnS_2_ and Cu/N‐SnS_2‐x_ i) Calculated work functions of SnS_2_ and Cu/N‐SnS_2‐x_.

Raman spectroscopy was utilized to validate the existence of Cu/N‐SnS_2‐x_. The signal at 312 cm^−1^, which corresponds to the A_1g_ mode of SnS_2_, indicated the presence of SnS_2_ (**Figure** **3**a).^[^ ^26^ ^]^Besides, the A_1g_ peak intensity was decreased due to the introduction of Cu and N. In this case, the introduction of N element is not obvious for the attenuation of the characteristic Raman peak of SnS_2_, while the introduction of Cu element plays an obvious attenuation of the characteristic peak, indicating that the introduction of the metal Cu element plays a key role in the attenuation of the SnS_2_ crystals. The electron spin resonance (ESR) method was further applied to confirm the existence of sulfur vacancies in Cu/N‐SnS_2‐x_. The ESR spectra (Figure 3b) revealed a detectable signal at a *g* value of 2.003, indicating unsaturated unpaired electrons caused by the existence of S vacancies. In detail, the ESR spectra of N‐SnS_2‐x_, Cu‐SnS_2‐x,_ and Cu/N‐SnS_2‐x_ all indicate the existence of S vacancies on their surface. However, N‐SnS_2‐x_ exhibits the lowest S vacancies concentration compared to Cu‐SnS_2‐x_ and Cu/N‐SnS_2‐x_, whereas the concentration of S vacancies increases dramatically when the metal Cu is introduced, suggesting that the metal element Cu is the key to increase the concentration of sulfur vacancies.^[^ ^27^ ^]^ X‐ray photoelectron spectroscopy (XPS) was used to determine the chemical characteristics of prepared materials. First, the XPS survey (Figure S4, Supporting Information) shows the existence of Cu, N, Sn, and S and the corresponding atomic ratios are 2.83at%, 3.72 at%, 21.8at%, and 39.78 at%, respectively. Figure 3c illustrates a detailed inspection of the Cu 2p XPS fine spectra, which verifies the presence of Cu 2p_3/2_ and Cu 2p_1/2_ due to the unique peaks found at 932.6 and 952.4 eV, respectively, implying Cu exists in Cu/N‐SnS_2‐x_.Besides, Auger electron spectroscopy (AES) was further applied to determine the existing state of Cu, the Cu LMM AES spectrum shows a distinct peak at 569 and 570 eV, contributing to the existence of Cu^2+^ and Cu^1+^. The ratio of Cu^2+^ and Cu^1+^ is 1(33%):2(67%), indicating the main valence state is Cu^1+^ and further proving the successful doping of Cu.^[^ ^28^ ^]^ Besides, as shown in Table S1 (Supporting Information), the ICP‐OES results indicate the percentage of Cu doping is 5.762wt.%. The N 1s XPS spectra and matching peak analyses of Cu/N‐SnS_2‐x_ are displayed in Figure 3d. The emergence of the N─Sn bond at 399.8 eV suggests that partial N atoms have replaced the location of S in SnS_2_ and heterogeneous N atoms have been successfully induced into SnS_2_.^[^ ^29^ ^]^ Sn^4+^ levels appear in the XPS spectra for the Sn 3d area for SnS_2_ and Cu/N‐SnS_2‐x_ at 486.6 eV (Sn3d_5/2_) and 495.1 eV (Sn3d_3/2_), respectively (Figure 3e).

Figure 3f illustrates how the S2p spectra for SnS_2_ and Cu/N‐SnS_2‐x_ exhibit the S 2p_3/2_ (161.6 eV) and S 2p_1/2_ (163 eV) states of S. The Sn 3d and S 2p spectra of Cu/N‐SnS_2‐x_ show a shift to lower binding energies compared to SnS_2_, which is noteworthy. This shift indicates a reduced valence state and an increased number of electrons in Cu/N‐SnS_2‐x_ due to the presence of sulfur vacancies.^[^ ^27^ ^]^

The electronic structures of SnS_2_ and Cu/N‐SnS_2‐x_ are further examined by DFT calculations. Detailed charge analysis (Figure 3g(A)–h(A)) reveals the charge exchange after the introduction of Cu and N. Compared to SnS_2_, Cu/N doping generates sulfur vacancies, which subsequently transfer electrons to neighboring Sn, increasing the charge of Sn. (0.836 |e| vs 0.912 |e| for Sn atom). The electron localization function (ELF) is utilized for assessing electron distribution in SnS_2_ and Cu/N‐SnS_2‐x_. As shown in Figure 3g(B)–h(B), Sulfur vacancies cause increased electron localization around the vacancy, resulting in non‐uniform electron distribution and charge transfer around metal atoms.^[^ ^30^ ^]^ Besides, the strategy of Cu/N doping can reduce the work function from 6.172 eV of SnS_2_ to 5.774 eV of Cu/N‐SnS_2‐x_. As a result, a lower work function of Cu/N‐SnS_2‐x_ could enhance charge transfer from the catalyst surface to the absorbed NO_2_^−^ and intermediates, which is favored for the NO_2_RR process.^[^ ^31^ ^]^

**Figure 4:**
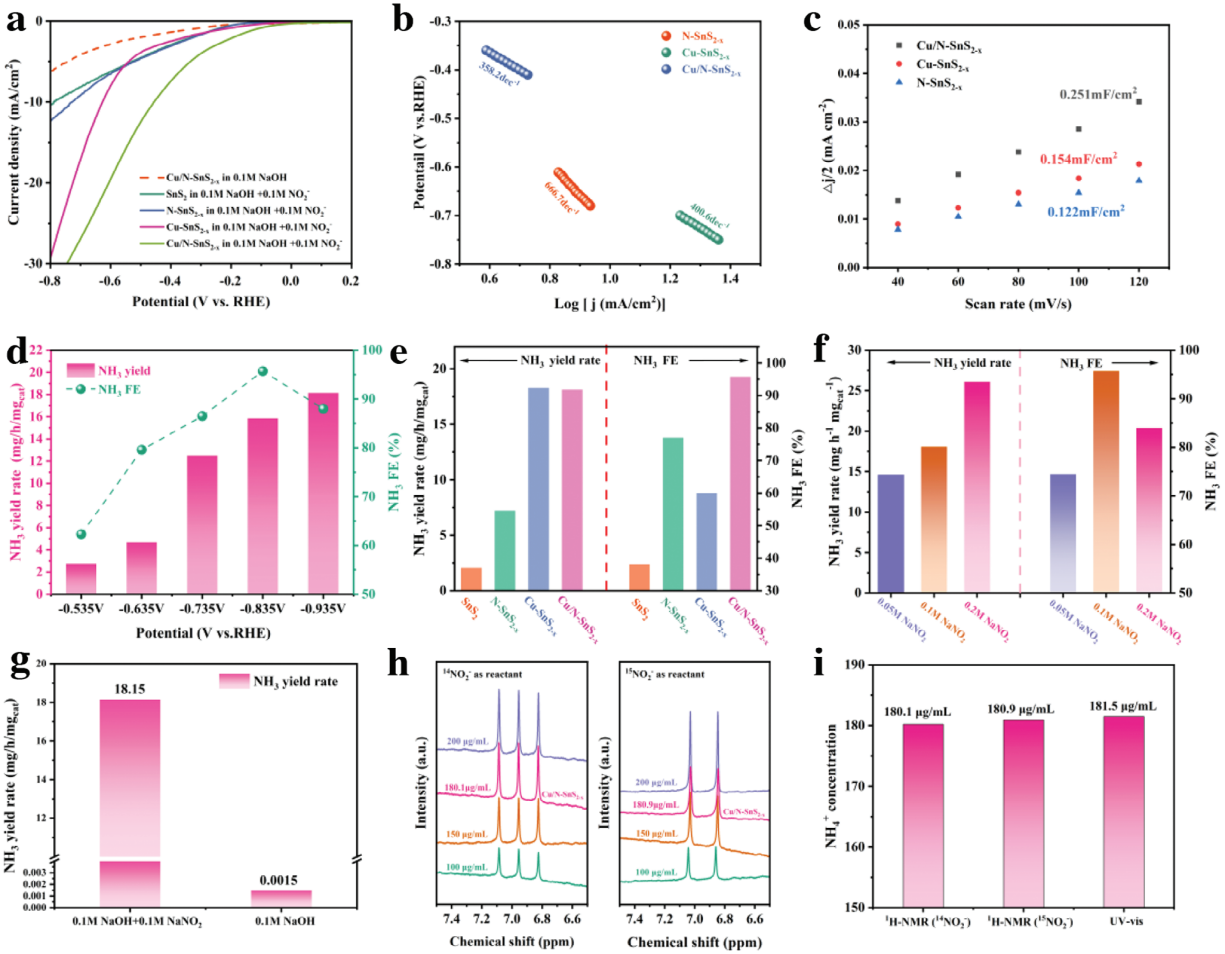
a) LSV curves b) Tafel plots. c) C_dl_ values. d) NH_3_ yields and NH_3_FEs of Cu/N‐SnS_2‐x_ at different potentials. e) NH_3_ yields rate and NH_3_ FE of SnS_2_, N‐SnS_2‐x,_ Cu‐SnS_2‐x_, and Cu/N‐SnS_2‐x_. f) NH_3_ yields rate and NH_3_ FE of Cu/N‐SnS_2‐x_ under various nitrite concentrations. g) Blank controls experiment h) ^1^H‐NMR measurements. i) Comparison NH_3_ yields of using different detection methods.

To investigate the electrochemical NO_2_^−^ reduction capabilities of Cu/N‐SnS_2‐x_, electrochemical measurements were tested at room temperature using a typical three‐electrodes system. Before the electrochemical test, cyclic voltammetry (CV) curves were measured until the polarization curves achieved a stable condition. First, electrochemical performance was assessed by linear sweep voltammetry (LSV).^[^ ^32^ ^]^ As shown in **Figure** **4**a, the LSV curve of Cu/N‐SnS_2‐x_ in 0.1 m NaOH with 0.1 m NaNO_2_ shows an obvious increase in current density compared with that without nitrite, indicating that nitrite was involved in the electrochemical reaction.^[^ ^33^ ^]^ Furthermore, LSV curves of N‐SnS_2‐x_ and Cu‐SnS_2‐x_ revealed the same trend, but both the current densities were much less than that of Cu/N‐SnS_2‐x_, which indicates that the co‐doping strategy of Cu and N could synergistically promote nitrite reduction rate. To further reveal the NO_2_RR enhancement observed in Cu/N‐SnS_2‐x_, Tafel plots were applied to reveal the catalytic activity of Cu/N‐SnS_2‐x_, N‐SnS_2‐x_, and Cu‐SnS_2‐x_. As shown in Figure 4b, the Tafel slope of Cu/N‐SnS_2‐x_ was ≈358.2 mV dec^−1^, which was significantly less than 666.7 mV dec^−1^ for N‐SnS_2‐x_ and 400.6 mV dec^−1^ for Cu‐SnS_2‐x_, indicating that Cu/N‐SnS_2‐x_ has relatively rapid kinetics for converting NO_2_^−^ to NH_3_.^[^ ^34^ ^]^ Besides, the double‐layer capacitance (C_dl_) was measured to determine the electrochemically active surface area (ECSA). As shown in Figure 4c, Cu/N‐SnS_2‐x_ (0.251 mF cm^−2^) presented a higher C_dl_ than N‐SnS_2‐x_(0.122 mF cm^−2^) and Cu‐SnS_2‐x_(0.154 mF cm^−2^), suggesting that Cu/N‐SnS_2‐x_ had more electrochemically active sites derived from the defective structure of Cu/N‐SnS_2‐x_ nanosheets which is favored for NO_2_RR.^[^ ^35^ ^]^As shown in Figure S5 (Supporting Information), impedance measurements of all four samples indicate the undoped SnS_2_ exhibits the largest charge transfer resistance, which may be caused by the semiconductor characteristic of SnS_2_ and its poor conductive properties also contribute to its poor catalytic properties. Whereas, with the introduction of N and Cu elements, the electrical conductivity of SnS_2_ has been enhanced and Cu/N‐SnS_2‐x_ exhibits a much smaller charge transfer resistance, suggesting Cu/N co‐doping and the introduction of S vacancies can significantly enhance the conductivity of SnS_2_.

To study the NO_2_RR activity of Cu/N‐SnS_2‐x_, chronoamperometry tests were performed at various applied potentials and the concentrations of NH_3_ were further quantified by UV–vis spectroscopy.^[^ ^36^ ^]^ As shown in Figure 4d, Cu/N‐SnS_2‐x_ exhibits an outstanding NO_2_RR performance with an FE of 95.73% at −0.835 V(vs RHE) and NH_3_ yield of 18.15mg h^−1^ mg_cat_^−1^ at −0.935 V(vs RHE), suggesting the Cu/N‐SnS_2‐x_ has excellent NO_2_RR performance, which is significantly superior to previous reported materials (Table S3, Supporting Information). Furthermore, the Faradaic Efficiency (FE) of Cu/N‐SnS_2‐x_ first increases and then decreases as the potential decreases, indicating that the FE of ammonia production is related to the applied potential, and this phenomenon can be explained by competition between intermediates containing N and H on the catalyst surface. As a comparison, N‐SnS_2‐x_ and Cu‐SnS_2‐x_ also tested NO_2_RR performance at the same potentials. As shown in Figure 4e, N‐SnS_2‐x_ and Cu‐SnS_2‐x_ show relatively inferior NH_3_ yield rates with 7.2 and 18.25 mg h^−1^ mg_cat_^−1^ and the highest FE of N‐SnS_2‐x_ and Cu‐SnS_2‐x_ are 77.13% and 60.32%, respectively, suggesting that the introduction of Cu/N and sulfur vacancy together improvs the NO_2_RR performance of SnS_2_. The NO_3_RR performance of Cu/N‐SnS_2‐x_ was also investigated. As shown in Figure S17c,d (Supporting Information), the NH_3_ yield rate and FE of Cu/N‐SnS_2‐x_ are significantly lower than NO_2_RR performance, suggesting NO_2_^−^ more suitable as raw material for electrochemical ammonia synthesis. In addition, NO_2_^−^ and NO_3_^−^ exist in the actual wastewater, thus wastewater simulation experiments are further applied. The composition of simulated wastewater that was selected is 7.1g L^−1^ Na_2_SO_4_; 3.5 g L^−1^ NaNO_3_·(40 mm);2.76 g L^−1^ NaNO_2_·(40 mm)and 0.7 g L^−1^ NaCl, and further adjust the initial pH to 13 with 0.1 m NaOH. As shown in Figure S19c (Supporting Information), the maximum ammonia yield in simulated wastewater reaches 5.97 mg h^−1^ mg_cat_^−1^ at the potential of −0.935 V, the ammonia yield gradually increases with the decrease of applied potentials. The highest FE in simulated real wastewater is 67% at −0.835 V (Figure S19d, Supporting Information). The lower FE may be due to the lower concentration gradient on the Cu/N‐SnS_2‐x_ surface due to the relatively low nitrate and nitrite concentration (40 mm), and the wastewater contains other heteroatoms, which limits the mass transfer and adsorption of nitrate and nitrite. To sum up, Cu/N‐SnS_2‐x_ can be used to eliminate excessive nitrate in industrial wastewater, but electrochemical reactor and operating conditions need to be optimized for future practical application.^[^ ^37^ ^]^

To evaluate the adaptability of Cu/N‐SnS_2‐x_ in varied nitrite concentrations, we conducted additional tests utilizing different NO_2_^−^ concentration gradients: 0.05m, 0.1 m, and 0.2m NaNO_2_. Figure 4 shows the LSV curve of Cu/N‐SnS_2‐x_ evaluated at different nitrite concentrations. Cu/N‐SnS_2‐x_ could reduce nitrite at varying nitrite concentrations, as evidenced by the current density's progressive increase with nitrite concentration. As shown in Figure 4f, the NH_3_ yield rates decrease gradually as the NO_2_^−^ concentration decreases and the NH_3_ yield rates are linearly dependent on the NO_2_^−^ concentration, suggesting that the electrochemical reduction of NO_2_^−^ to NH_3_ is a first‐order reaction with respect to the NO_2_^−^ concentration. The FEs also demonstrate a downward trend with decreasing NO_2_^−^ concentration when the concentration of NO_2_^−^ is decreased to 0.05m, Cu/N‐SnS_2‐x_ still delivers a high FE of 74.52% indicating that Cu/N‐SnS_2‐x_ has a potential to be applied to large‐scale industrial production.^[^ ^38^ ^]^

Blank control experiments were further conducted to rule out the possibility of N contamination from the external environment. The extremely low yield of NH_3_ in the blank electrolyte (Figure 4g) demonstrates that the N of NH_3_ was derived from NO_2_^−^.^[^ ^39^ ^]^ To further verify the N origin of the generated NH_3_, the ^15^N isotope labeling experiment combined with the ^1^H nuclear magnetic resonance (^1^H‐NMR) technology is undoubtedly the best‐guaranteed method. A set of quantitative ^1^H‐NMR tests was conducted to prove the authenticity and reliability and according to ^1^H‐NMR spectra of Figure 4h showing the different concentrations of ^14^NH_4_^+^ and ^15^NH_4_^+^, the calculated ^14^NH_4_^+^ and ^15^NH_4_^+^ concentrations of Cu/N‐SnS_2‐x_ at −0.935V vs RHE is 180.1 and 180.9µg mL^−1^, respectively (181.5µg mL^−1^ using UV–vis) after integrating the obtained peak areas, which further confirming the accuracy of determining NH_3_ yield rate (Figure 4i). As shown in Figure S18 (Supporting Information), the ^1^H‐NMR spectra of the electrolyte after electrocatalysis using Na^15^NO_2_ as a reactant also showed two peaks corresponding to ^15^NH_4_^+^ and the ^1^H‐NMR spectrum revealed typical triple peaks corresponding to ^14^NH_4_^+^ while Na^14^NO_2_ was used as electrolyte.^[^ ^40^ ^]^ This result bears out the fact that the formation of NH_3_ derives from the NO_2_^−^ electroreduction.

**Figure 5:**
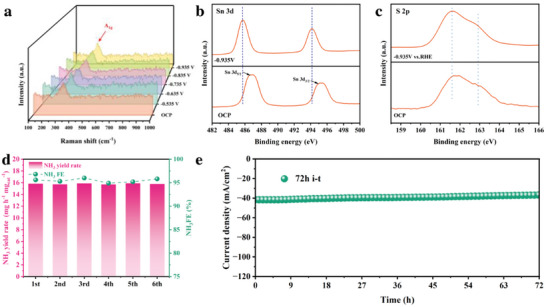
a) In situ Raman test. In situ XPS results of Cu/N‐SnS_2‐x_ under −0.935V b) XPS Sn3d spectra. c) XPS S2p spectra. d)NH_3_ yields rate and FEs of six‐times recycle test. e) 72 h *i‐t* test.

Stability is a crucial benchmark to estimate catalysts for potential practical applications. In situ Raman spectroscopy was applied to optimize the structure stability of Cu/N‐SnS_2‐x_ during the reaction. As shown in **Figure** **5**a, when applied potential decreases from −0.535 to −0.935 V, the significant A_1g_ vibration peak of SnS_2_ remains unchanged at 312 cm^−1^ during the electrolysis process, indicating the main structure of SnS_2_ sustains its stability. Besides, in situ XPS was further applied. As shown in Figure 5b,c, the in situ XPS test results of Sn and S show that there is no obvious peak change between Sn and S during the NO_2_RR process, especially for Sn, there is no existence of Sn^2+^ and Sn^0^ during the reaction, which indicates that the main structure of SnS_2_ is stable. The cyclic stability of Cu/N‐SnS_2‐x_ was shown in Figure 5d under optimal NH_3_ selectivity reaction conditions. Throughout the recycling trials, the NH_3_ yield rate and NH_3_ FE were maintained during the cycling tests, highlighting the exceptional stability of Cu/N‐SnS_2‐x_ in NO_2_RR applications. Moreover, a continuous electrolysis lasting 72 h *i‐t* test was performed using an H‐cell reactor as shown in Figure 5e, indicating the remarkable stability of Cu/N‐SnS_2‐x_. As shown in Figure S27 (Supporting Information), the EIS result of Cu/N‐SnS_2‐x_ after the NO_2_RR test showed an infinitesimal change compared to the EIS of Cu/N‐SnS_2‐x_ before the NO_2_RR test, which further proves the excellent stability of Cu/N‐SnS_2‐x_ during the NO_2_RR process. XRD tests (Figure S30, Supporting Information), TEM image (Figure S31, Supporting Information), HRTEM image(Figure S32, Supporting Information), and XPS results (Figure S33, Supporting Information) of Cu/N‐SnS_2‐x_ after 72h *i‐t* test show excellent structure stability of Cu/N‐SnS_2‐x_. Collectively, the post‐test characterization data suggests that Cu/N‐SnS_2‐x_ is chemically stable enough for NO_2_RR.

**Figure 6:**
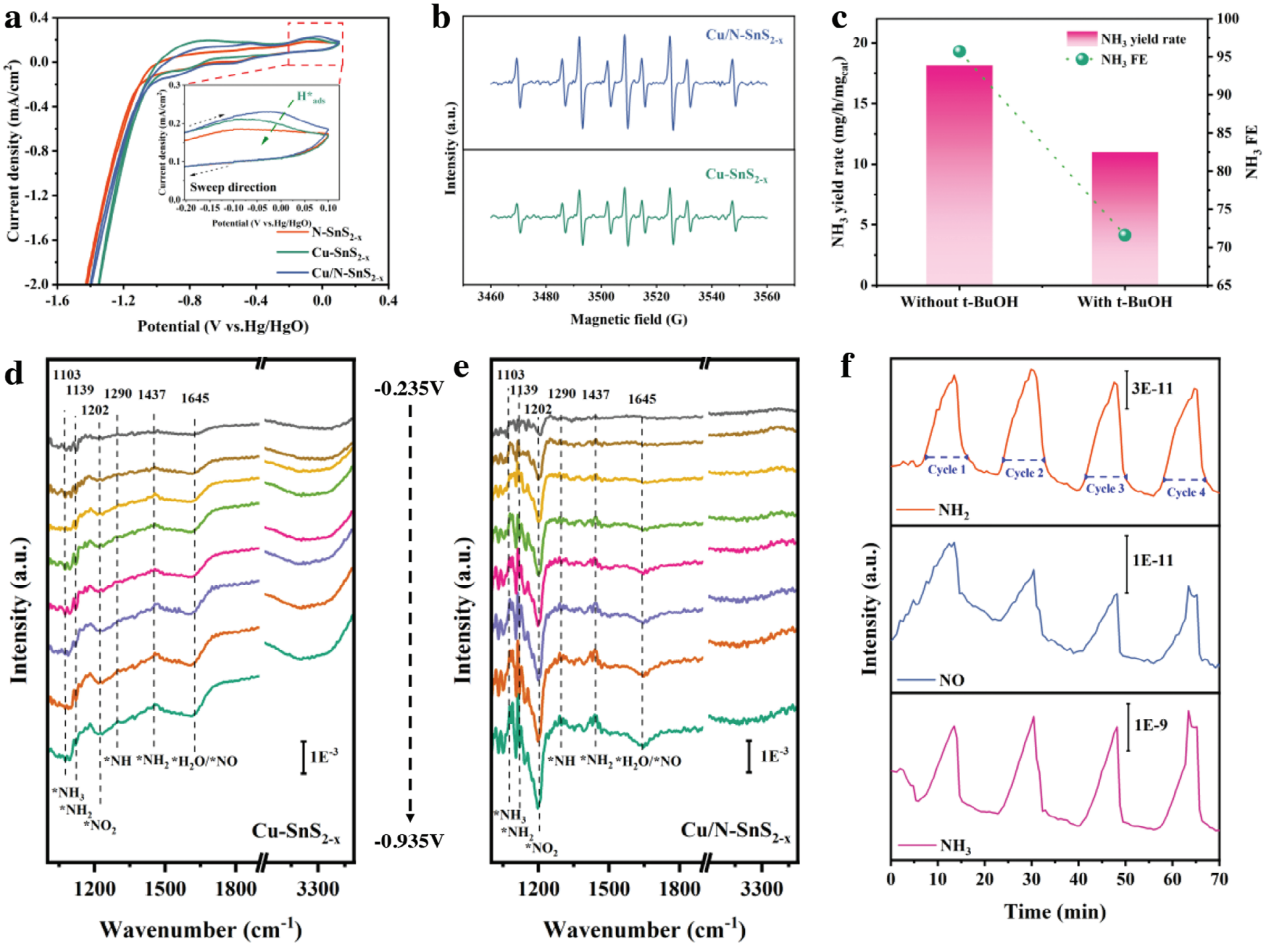
a) Cyclic voltammetry curves of N‐SnS_2‐x,_ Cu‐SnS_2‐x_, and Cu/N‐SnS_2‐x_. b) Electron spin resonance spectra of Cu‐SnS_2‐x_, and Cu/N‐SnS_2‐x_. c) NH_3_ yields rate and FEs of Ni‐ Cu/N‐SnS_2‐x_ with and without *t*–BuOH.d) In situ FT‐IR spectra of Cu ‐SnS_2‐x_ at different potentials for NO_2_ RR. e) In situ FT‐IR spectra of Cu/N‐SnS_2‐x_ at different potentials for NO_2_ RR. f) DEMS spectra of Cu/N‐SnS_2‐x_ at different potentials for NO_2_ RR. d) PDOS of SnS_2_ e) PDOS of Cu/N‐SnS_2‐x_ f) Adsorption patterns of nitrite on SnS_2_ and Cu/N‐SnS_2‐x_ surfaces.

In order to further explore the role of a co‐doping strategy for enhanced NO_2_RR performance, a set of characterizations was applied. First, a cyclic voltammetric test was applied to different samples to evaluate their ability to generate atomic H^*^, which is an essential component for the following nitrite reduction. The cyclic voltammetric test was applied at the potential range from 0.1 to −1.5V (vs Hg/HgO) in 0.1m NaOH + 0.1m NaNO_2_, the characteristic peaks of H^*^ appear at 0–−0.15 V (vs Hg/HgO), suggesting the atomic H^*^ was generated during the NO_2_RR process and involved in the conversion process of nitrite (**Figure** **6**a).^[^ ^41^ ^]^ Obviously, Cu/N‐SnS_2‐x_ exhibits a higher intensity of H_ads_^*^ than Cu‐SnS_2‐x_, indicating the introduction of N enhanced the ability to generate active hydrogen (H_ads_^*^) which is beneficial for nitrite reduction. Furthermore, we conduct ESR measurements using 5,5‐dimethyl1‐pyrroline‐N‐oxide (DMPO) as the H_ads_^*^ trapping reagent to assess the amounts of H_ads_^*^ produced or consumed during the NO_2_RR electrolysis.^[^ ^42^ ^]^ As shown in Figure 6b, ESR spectra reveal strong DMPO‐H signals for both Cu/N‐SnS_2‐x_ and Cu ‐SnS_2‐x_ after electrolysis in 0.1m NaOH, implying the strong H_2_O dissociation capability of Cu/N‐SnS_2‐x_ and Cu‐SnS_2‐x_ to produce abundant ^*^H_ads_ and Cu/N‐SnS_2‐x_ exhibited much stronger ESR intensity than that of Cu‐SnS_2‐x_, suggesting that the introduction of elemental N further facilitates the promotion of the ability to produce H_ads_^*^, which contributes to the successful conduct of the nitrite reduction reaction.^[^ ^30^ ^]^ Besides, we used tertiary butanol (*t*–BuOH) to trap ^*^H, and the NO_2_RR performance comparison is shown in Figure 6c. When *t*–BuOH is added, the NO_2_RR performance of Cu/N‐SnS_2‐x_ is obviously weakened, indicating that the formation of ^*^H can promote the NO_2_RR process.

In situ attenuated total reflectance surface‐enhanced infrared absorption spectroscopy (ATR‐SEIRAS) was also applied to detect the reaction intermediates adsorbed on Cu‐SnS_2‐x_ (Figure 6d) and Cu/N‐SnS_2‐x_ (Figure 6e) surface during the NO_2_RR process. When potential decreases from −0.235 to −0.935 V, several peaks appear and increase in intensity in both Cu‐SnS_2‐x_ and Cu/N‐SnS_2‐x_. Signals were generated at wavenumbers of 1103, 1139, 1202, 1290, 1437, and 1645 cm^−1^, which respectively represented NH_3_, NH_2_, NO_2_, NH, NH_2_, and NO adsorbed on the surface of Cu‐SnS_2‐x_ and Cu/N‐SnS_2‐x_ during the reaction, indicating NO_2_^−^ effectively transformed to NH_3_ through a multiple electron transfer process.^[^ ^43^ ^]^ However, the signal intensity on the Cu/N‐SnS_2‐x_ surface is much stronger, indicating the Cu─N co‐doping strategy can effectively enhance the process of transformation of NO_2_^−^.In addition, the generation of various H‐containing intermediates indicates that atomic hydrogen (^*^H_ads_) generated in Cu/N‐SnS_2‐x_ is further applied to the hydrogenation of NO_2_^−^, which effectively promotes the electrochemical reduction of NO_2_^−^ to NH_3_. Online differential electrochemical mass spectrometry (DEMS) experiments of Cu/N‐SnS_2‐x_ were further applied to detect the intermediates and gas products generated during the NO_2_RR (Figure 6f). Under the potential of −0.935 V vs RHE, Cu/N‐SnS_2‐x_ produces other gas products in addition to NH_3_ (17), including *m*/*z* signals of NO (30), and NH_2_ (16) suggesting that the conversion of nitrite to ammonia indeed occurred on Cu/N‐SnS_2‐x_ surface.

To understand the enhanced NO_2_RR performance of Cu/N‐SnS_2‐x_ caused by dual element doping strategy, density functional theory (DFT) calculations were performed.

**Figure 7:**
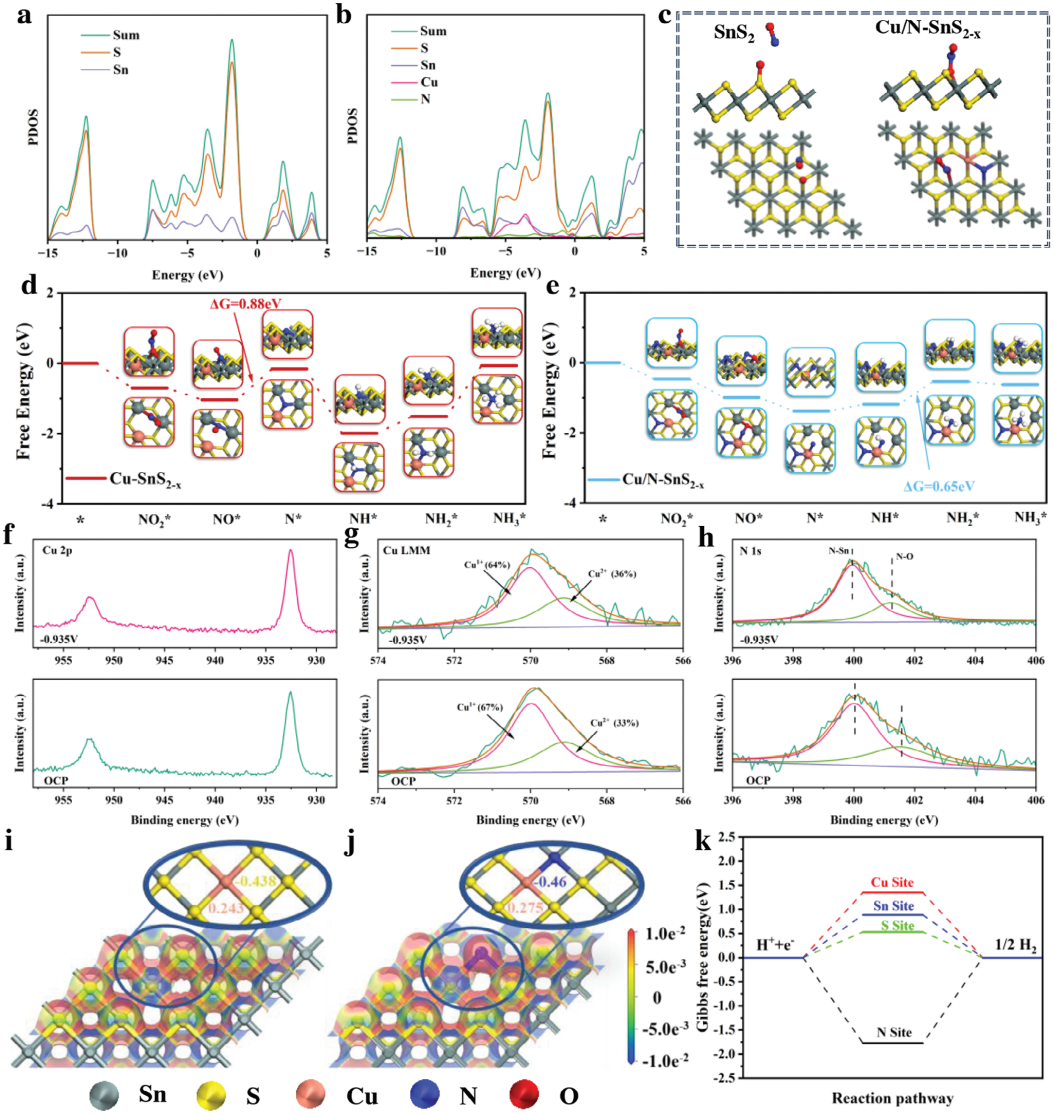
a) PDOS of SnS_2_ b) PDOS of Cu/N‐SnS_2‐x_ c) Adsorption patterns of nitrite on SnS_2_ and Cu/N‐SnS_2‐x_ surfaces. d) Calculated free energy changes of nitrite reduction reaction on Cu/N‐SnS_2‐x_. e) Calculated free energy changes of nitrite reduction reaction on Cu ‐SnS_2‐x_. In situ XPS results of Cu/N‐SnS_2‐x_ under −0.935V f) XPS Cu2p spectra g) Cu LMM AES spectra. h) XPS N 1s spectra. i,j) Charge density difference of Cu‐SnS_2‐x_ and Cu/N‐SnS_2‐x_ k) Gibbs free energy of hydrogen on different sites of Cu/N‐SnS_2‐x_.

We explore the effect of sulfur vacancies and elemental doping on the conductivity of SnS_2_ by calculating the Projected density of states (PDOS) of the relevant samples. First, as shown in **Figure** **7**a, the PDOS analysis indicates that SnS_2_ possesses a tangible bandgap indicative of its semiconducting character, suggesting its poor electrical conductivity, which is consistent with the EIS results. In contrast, as S vacancy and Cu─N was introduced, SnS_2‐x_ (Figure S21, Supporting Information) and Cu/N‐SnS_2‐x_ (Figure 7b) exhibit noticeable electronic states crossing the Fermi level, leading to the metallic characteristics of SnS_2‐x_ and Cu/N‐SnS_2‐x_, thus higher conductivity relative to that of SnS_2_, which is favorable for the proton‐coupled electron‐transfer process to boost the NO_2_RR kinetics. Furthermore, we performed the corresponding calculations for the adsorption of nitrite on the SnS_2_ and Cu/N‐SnS_2‐x_ surfaces. As shown in Figure 7c, NO_2_^−^ cannot efficiently adsorb on the SnS_2_ surface, due to the complete encapsulation of the inner metal sites and the inability of the outer S atoms to efficiently adsorb NO_2_^−^. As for Cu/N‐SnS_2‐x_, the S vacancies created by Cu/N doping effectively expose the internal metal sites. The exposed Sn sites effectively form an adsorption structure with NO_2_^−^, providing a prerequisite for the subsequent reduction reaction.

In order to investigate the reaction pathways for the reduction of nitrite to ammonia, we calculated the Gibbs free energy diagram for the reduction reaction of nitrite on Cu/N‐SnS_2‐x_ (Figure 7d) and Cu‐SnS_2‐x_ (Figure 7e) surfaces, respectively. In detail, nitrite can be successfully transferred into NH_3_ on Cu‐SnS_2‐x_ and Cu/N‐SnS_2‐x_ surfaces, including the corresponding reaction intermediates (NO_2_^*^, NO^*^, N^*^, NH^*^, NH_2_^*^, NH_3_^*^), suggesting the doping strategy is an effective method to enhance the NO_2_^−^ reducing abil of SnS_2_. From the Gibbs free energy diagram, it is clear that the rate‐limiting step for NO_2_^−^ reduction on Cu‐SnS_2‐x_ surface is the formation of N^*^ intermediate, which requires an energy input of 0.88 eV. Besides, the formation of N^*^ intermediate on Cu‐SnS_2‐x_ surface involved the conversion between metal sites, in which Cu atoms effectively adsorb with N and participate in the subsequent reduction reaction, suggesting that Cu is the crucial active site during the NO_2_RR process. The rate‐limiting step for Cu/N‐SnS_2‐x_ is the formation of the NH_2_^*^ intermediate which requires an energy input of 0.65 eV which is lower than the rate‐limiting step of Cu‐SnS_2‐x_, indicating nitrite is much easier to reduce on Cu/N‐SnS_2‐x_ surface. Besides, due to the further introduction of nitrogen, the Cu site can directly form an adsorption structure with the NO^*^ intermediate in the early stage of the reduction reaction, which participates in the process of the reduction reaction and reduces the reaction energy barrier of the nitrite reduction reaction.

In order to further investigate the role of elemental N doping, the in situ XPS was applied to explore the charge transfer between Cu and N active sites during the NO_2_RR process. As shown in Figure 7f, Cu XPS results between the applied potential of OCP and −0.935 V vs RHE suggest that Cu2p has not changed significantly and Cu is still 1 + dominant, while the further comparison of the Cu LMM orbitals also reveals that the ratio of Cu^2+^ is slightly increased to 36%, indicating that Cu will further lose electrons during the NO_2_RR process, leading to its electron‐deficient state. The XPS results of elemental N show that the overall peak position of N is shifted to the lower binding energy, indicating that N has gained electrons during the reaction process and is in an electron‐rich state, which may be due to its attraction of electrons from the neighboring Cu. Besides, we performed differential charge density calculations for Cu‐SnS_2‐x_ and Cu/N‐SnS_2‐x_, respectively. As shown in Figure 7i,j, it is clearly found that after N doping, due to the electron‐absorbing property of N, the electrons are further transferred from Cu to N, which makes Cu in an electron‐deficient state. This characteristic makes Cu more favorable to complete the adsorption process of the reaction intermediates, which reduces the corresponding reaction energy barrier and is conducive to the smooth progress of the NO_2_^−^ reduction reaction. Furthermore, the hydrogen formation free energy was further investigated on different sites in Cu/N‐SnS_2‐x_. As shown in Figure 7k, the hydrogen formation free energy on S site is 0.5304 eV, which is much closer to 0 eV compared to Cu sites (1.3483 eV), Sn sites (0.886 eV) and N (−1.772 eV), indicating hydrogen is more likely to adsorb on S sites rather than other active sites on Cu/N‐SnS_2‐x_.^[^ ^35^ ^]^ Besides, the calculation of the adsorption of OH^−^ on different active sites is shown in Figure S35 (Supporting Information), the adsorption energies of t N and S for OH^−^ are both positive, which indicates that these two elements have poor adsorption capacity for OH^−^, while the adsorption energies of the metallic elements Cu and Sn for OH^−^ are both negative, which indicates that OH^−^ can be adsorbed on the metal active sites. Besides, a specific comparison of the adsorption energies shows that the adsorption capacity of Cu for OH^−^ is stronger than Sn, indicating that OH^−^ will prefer to adsorb on the Cu site. Comparing the adsorption energy of NO_2_^−^, it can be seen that NO_2_^−^ is preferentially adsorbed with Sn atoms on the catalyst surface and the adsorption energy is significantly lower than that of OH^−^, which indicates that Sn also reacts with NO_2_^−^ preferentially in the reaction process. This phenomenon facilitates the liberation of metal sites for the preferential adsorption of nitrite and subsequent reactions, further promoting the efficient synthesis of ammonia from nitrite.

Collectively, the enhanced NO_2_RR performance of Cu/N‐SnS_2‐x_ can be attributed to the following points. First, the S vacancies formed due to the doping process effectively expose the internal metal sites and are further used for NO_2_^−^ adsorption and activation. Second, the doped N element can effectively transfer the electrons from Cu to N, make Cu turn into electron‐deficient state and effectively promote the adsorption of Cu to the intermediates of the NO_2_RR process and reduce the reaction energy barrier. Third, the doped N element effectively enhances the active hydrogen generation capacity of Cu/N‐SnS_2‐x_, which is favorable to the hydrogenation process with nitrite and enhances its nitrite reduction rate.

## 3 Conclusion

In summary, sulfur vacancies rich Cu‐N co‐doped SnS2 nanosheet was successfully synthesized by hydrothermal method. Due to the co‐doped strategy, Cu and N were successfully introduced into SnS_2_ and facilitated the microenvironment in the van der Waals gap which can enhance the adsorption of NO_2_^−^ and the generation of H_ads_. Theoretical calculations revealed the introduced Cu and N could form the unique electron transfer pair leading Cu in the electron‐deficient state promoting the adsorption of nitrogen‐containing intermediates and reducing the energy barrier for nitrite reduction. Benefit from this, Cu─N co‐doped SnS_2_ achieves the highest NH_3_ yield rate of 18.15mg h^−1^ mg_cat_^−1^ at −0.935 V(vs RHE) and an excellent Faradaic Efficiency of 95.7% at −0.835 V(vs RHE) which are superior to others Sn‐based electrocatalysts. Our work not only offers a highly active and durable catalyst for NO_2_RR for NH_3_ generation but also paves the opportunity for the construction of transition metal sulfide catalysts with high catalytic performance.

## Conflict of Interest

The authors declare no conflict of interest.

## Supporting information



Supporting Information

## Data Availability

The data that support the findings of this study are available from the corresponding author upon reasonable request.
