# Supplementary material for: Modifying Microenvironment in Van der Waals Gap by Cu/N Co‐Doping Strategy for Highly Efficient Nitrite Reduction to Ammonia
Source: Adv Sci (Weinh). 2025 Feb 24;12(15):2417773. doi: 10.1002/advs.202417773 (PMC12005774; doi:10.1002/advs.202417773)
Supplement: Supplementary file 1 — Supporting Information [file ADVS-12-2417773-s001.docx]

**Modifying Microenvironment in van der Waals Gap by Cu/N Co-doping Strategy for Highly Efficient Ntrite Reduction to Ammonia**

Heen Li ^a^, Yuanzhe Wang ^a^, Kuo Wei ^a^, Maoyue He ^b^, Mengmeng Yan ^b^ ,Fei Peng ^c^, Faming Gao* ^a,b^

1. Tianjin Key Laboratory of Multiplexed Identification for Port Hazardous Chemicals, Tianjin University of Science & Technology, Tianjin 300222, P. R. China
2. Key Laboratory of Applied Chemistry, Yanshan University, Qinhuangdao 066004, P. R. China
3. Analyses and Testing Center, Hebei Normal University of Science and Technology, Qinhuangdao 066000, P. R. China

Corresponding Author

*Faming Gao: E-mail: [fmgao@tust.edu.cn](https://mail.tust.edu.cn/coremail/XT3/oab/userdetail.jsp?sid=BAPMjREECIBzNBgGrSEENIyhpFgaifGS&urlfrom=..%2foab%2flist.jsp%3fsid%3dBAPMjREECIBzNBgGrSEENIyhpFgaifGS%26dn%3da%252f22&dn=a%2f22&uid=fmgao@tust.edu.cn)

**Supporting information**

**Characterizations.**

The microtopography of the prepared samples was characterized by transmission electron microscopy (TEM, JEOL 2100 plus + ARM 200 F). Energy dispersive X-ray spectroscopy attached to the transmission electron microscope was used to obtain elemental composition. Raman spectroscopy (WITec, alpha300R, excited by a 512 nm laser) was also performed. The crystalline phases were performed by X-ray diffraction (XRD) using an Rigaku D/MAX-2500 powder diffractometer. The chemical states of the prepared sample were conducted using a Thermo Scientific ESCALAB 250Xi photoelectron spectrometer. The absorbance data of spectrophotometer were collected on a SHIMADZU UV-2550 Ultra-Violet visible (UV-vis) spectrophotometer. Electron spin resonance (ESR) measurements were performed using the Bruker ER 200D spectrometer at room temperature.

**Preparations of materials**

**Synthesis of Cu/N-SnS_2-x_**

Copper-nitrogen co-doped SnS_2_ nanoflowers were synthesized by a hydrothermal method. Firstly, 135 mg of tin dichloride (SnCl_2_) and 135 mg of thioacetamide (C_2_H_5_NS) were sequentially added into 25 mL of ethanol and stirred thoroughly for 20 min to form transparent solution, and then 50.45 mg of cyano-guanidine (C_2_H_4_N_4_) was continued to be added into the solution and stirred continuously for 20 min to form a transparent solution. Finally, 25.5 mg of copper chloride was added to the above transparent solution and stirred continuously for 20 min to form a light blue transparent solution, after which the solution was transferred to a 50 mL PTFE reactor and heated to 180 °C at a rate of 5 °C/min, and the reaction was allowed to cool naturally for 12 h. The dark brown product obtained was washed with anhydrous ethanol and centrifuged for three times, and then placed in an oven at 80 °C for one night after drying. The product was collected and recorded as Cu/N-SnS_2-x._

**Synthesis of N-SnS_2-x_**

The preparation of nitrogen doped SnS_2_ nanoflowers removes the incorporation of copper chloride as compared to the preparation of Cu/N-SnS_2-x_ nanoflowers. The other preparation methods were identical.

**Synthesis of Cu-SnS_2-x_**

The preparation of Cu-doped SnS_2_ nanoflowers removed the addition of cyano-guanidine as compared to the preparation of Cu/N-SnS_2-x_ nanoflowers. The other preparation methods were identical.

**Synthesis of Pristine-SnS_2_**

The preparation of Pristine-SnS_2_ nanoflowers removed the addition of cyano-guanidine and copper chloride as compared to the preparation of Cu/N-SnS_2-x_ nanoflowers. The other preparation methods were identical.

**Electrochemical experiment**

An ultrasonic mixer was used for three hours to distribute 10 mg of catalyst powder into 1.95 mL of ethanol and 50 μL of Nafion solution (5.0 wt%). All catalyst ink was created using this method. Carbon cloth (1.2 cm × 1 cm) was treated by immersing it in concentrated HNO_3_ at 70 °C for one hour. Following acid washing, the CC was ultrasonically treated for 0.5 hours in ethanol, acetone, and clean water. In preparation for the upcoming experiment, 50 microliters of homogeneous catalyst ink were vacuum-dried over well-treated CC (loading mass: 0.25 mg).

The electrochemical measurements were performed in an H-type electrolytic cell separated by a nafion 117 membrane at ambient temperature and standard atmospheric pressure (101kpa) using a CHI 660e electrochemical workstation (Chenhua, Shanghai). The working electrode surface area was limited to 1cm^2^. The cathode and anode compartments received an equal amount of 0.1 M NaOH (25 mL). For NO_2_^-^ reduction, 0.1M NaNO_2_ was added to the cathode compartment. The reversible hydrogen electrode (RHE) was used to record all potentials. Curves of linear sweep voltammetry (LSV) are measured at a rate of 5 mVs^-1^. Chronoamperometry test was performed for 1 hour at various potentials with a stirring rate of 500 rpm.

**Quantification of ammonia**

When tested in alkaline solution, we used spectrophotometry method to detect the quantification of ammonia. Briefly, after 1h electrocatalysis, 0.02 ml electrolyte was removed from cathode and diluted to 2 mL to detection range and following 2mL of 1M NaOH solution containing 5% salicylic acid and 5% sodium citrate was added into the solution. Subsequently, 1 mL of 0.05M NaClO and 0.2 mL of 1% C_5_FeN_6_Na_2_O·2H_2_O were add into the above solution. Then the solution was incubated under dark conditions at for 2h before UV-vis absorption spectrum was measured at a wavelength of 655 nm (Shimadzu, UV-2550). NH_4_^+^ calibration curve was calculated by using a series of different concentrations standard NH_3_ Solution (0μg/mL,0.25μg/mL,0.5μg/mL,0.75μg/mL,1μg/mL).NH_4_Cl was dried in oven before used. Calibration curve showed good linear relationship (y=0. 415x+0.054 R^2^=0.999).

**Calculation of the Faradaic efficiency and yield.**

The Faradaic efficiency of NO_2_RR was calculated as follows

FE = 6F × c × V / (17 × Q)

where F is the Faraday constant, c is the measured NH_3_ concentration, V is the volume of the electrolyte, and Q is the quantity of electric charge for one electron of NO_2_RR testing.

The Faradaic efficiency of NO_3_RR was calculated as follows

FE = 8F × c × V / (17 × Q)

where F is the Faraday constant, c is the measured NH_3_ concentration, V is the volume of the electrolyte, and Q is the quantity of electric charge for one electron of NO_3_RR testing.

The NH_3_ formation rate was determined using the following equation:

r(NH_3_) = (c × V)/(t × m)

where c is the measured NH_3_ concentration, V is the volume of the electrolyte , t is the reduction reaction time, and m is the loading mass of the sample (loading mass：0.25mg).

**Na^15^NO_2_ isotope labelling experiments**

Isotopically labeled nitrite reduction experiments were conducted to elucidate the ammonia source and quantify the concentration of ammonia using Na^15^NO_2_ (99%) as the feed N source. After electrolyzing a 0.1 M NaOH solution containing Na^15^NO_2_ (0.1M NaOH) for 2 h, electrolyte with ^15^NH_4_^+^ was extracted and the pH was further adjusted to weak acid by adding 4M H_2_SO_4_.Next, a 50 μL DMSO-d_6_ was blended with 1mL of acidified electrolyte and the further ^1^H NMR spectra was acquired via NMR analysis.

**Calculation details**

All calculations in this work were performed using the Vienna ab initio simulation package (VASP) based on the density functional theory (DFT). The projected augmented wave (PAW) method with PBE functional was employed for the generation of pseudopotential. The kinetic energy cutoff for the plane-wave expansion was set to 400 eV. The 3×3×1 k-point mesh set was used for all slab models. All the structural models were fully relaxed to the ground state with the convergence of energy and forces setting to 10^-5^ eV and 0.01 eV Å^-1^, respectively. SnS_2_ (001) was modeled by a 4×4 supercell and a vacuum space of around 20 Å was set along the z-direction.

Here, the chemical reaction considered can be summarized with the reaction equations below.

*+ NO_2_^-^ →*NO_2_ + e^-^
*NO_2_ + 2H^+^ + 2e^-^ →*NO + H_2_O
*NO + 2H^+^ + 2e^-^ →*N + H_2_O
*N + H^+^ + e^-^ → *NH
*NH + H^+^ + e^-^ →*NH_2_

*NH_2_+ H^+^ + e^-^→ *NH_3_

*NH_3_ → NH_3_ + *

where * represents the active site. Then, the reaction free energy change can be obtained with the equation below:
 Δ*G* = Δ*E* + Δ*E*_ZPE_ – *T*Δ*S*

where ΔE is the total energy difference before and after intermediate adsorbed, ΔE_ZPE_ and ΔS are, respectively, the differences of zero-point energy and entropy. The zero point energy and entropy of free molecules and adsorbents were obtained from the vibrational frequency calculations.

**Fig. S1 SEM image (a) and TEM image (b) of pristine-SnS_2_**

**Fig. S2 SEM image (a) and TEM image (b) of N-SnS_2-x_**

**Fig. S3 SEM image (a) and TEM image (b) of Cu-SnS_2-x_**

**
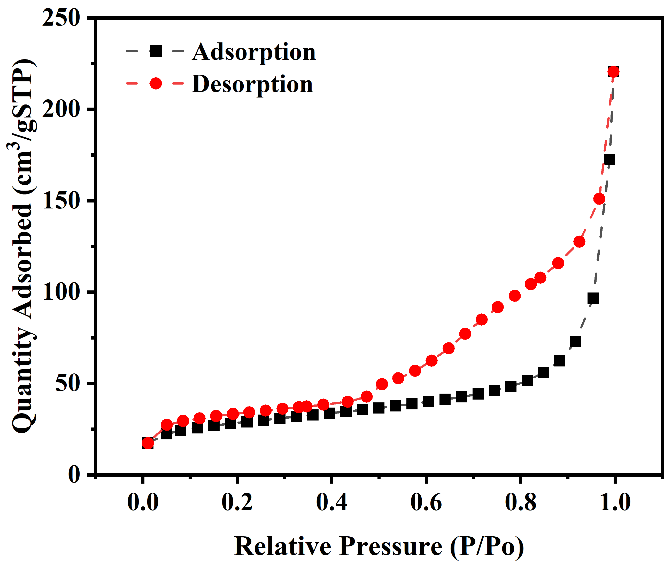
**

**Fig.S4 BET curve of Cu/N-SnS_2-x_**

**Fig.S5 The optimisation model of SnS_2_**

**Fig.S6 The optimisation model of Cu/N-SnS_2-x_**


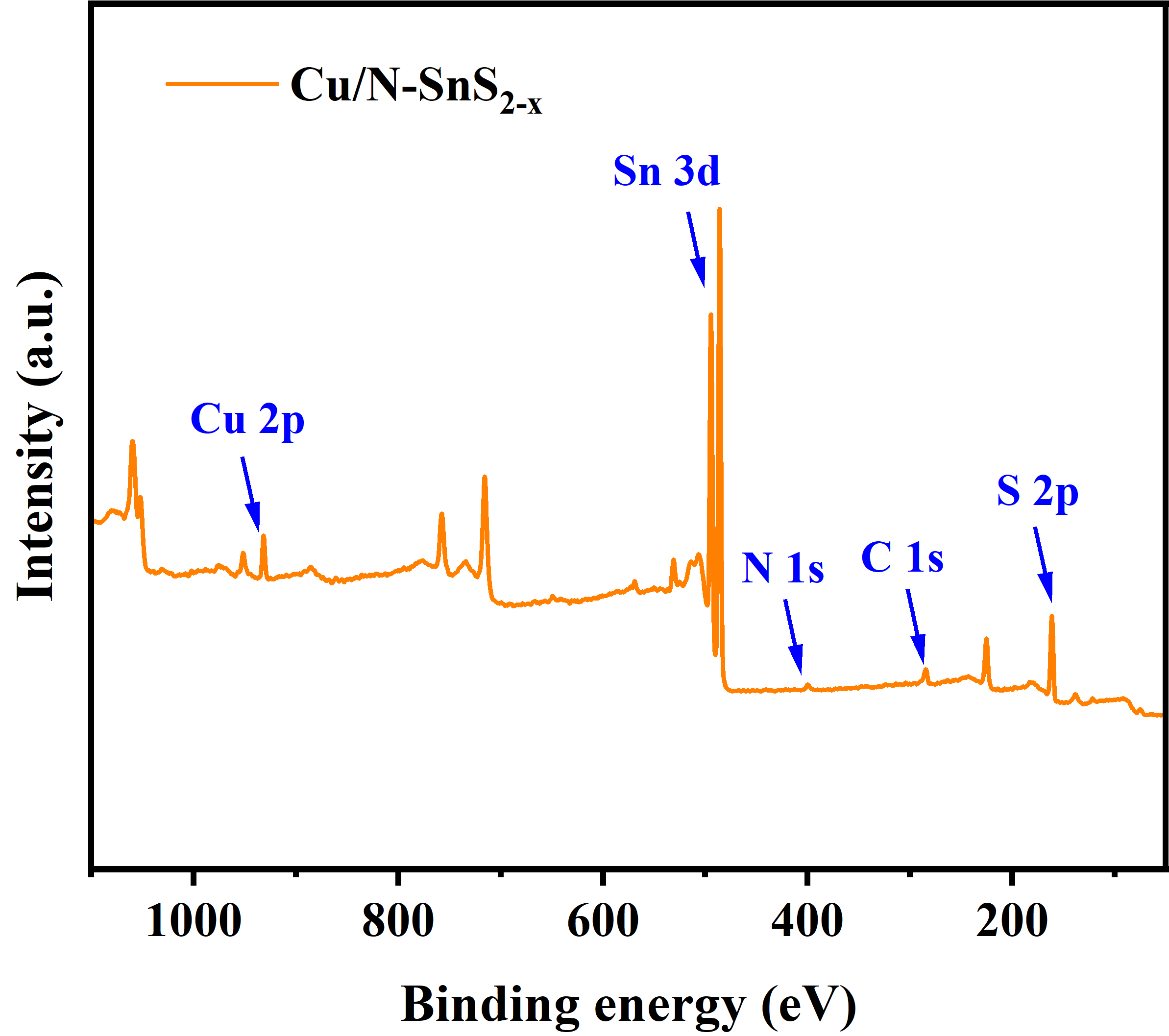


**Fig. S7 XPS survey of Cu/N-SnS_2-x_**

**
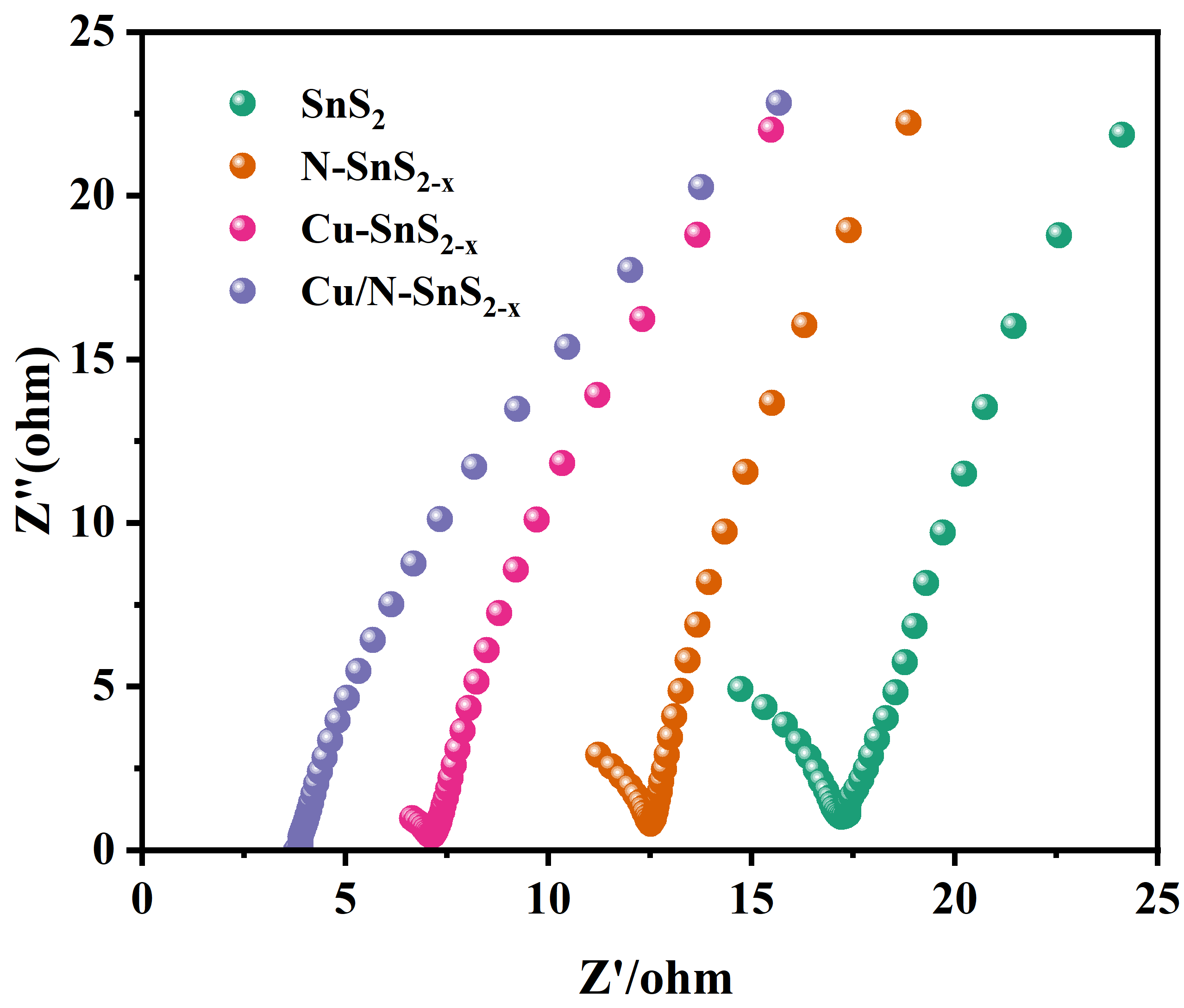
**

**Fig.S8 EIS tests of all samples**

**Fig. S9. CV curves of Cu/N-SnS_2-x_**

**Fig. S10. CV curves of N-SnS_2-x_**

**Fig. S11. CV curves of Cu-SnS_2-x_**

**Fig. S12. (a) UV-Vis spectra of various NH_3_ concentrations after incubated for 1 h at room temperature. (b) Calibration curve used for calculation of NH_3_ concentrations.**

**Fig. S13. Chronoamperometry curves of Cu/N-SnS_2-x_ at different potentials.**

**Fig. S14.** **UV-vis spectra of Cu/N-SnS_2-x_ at different potentials.**


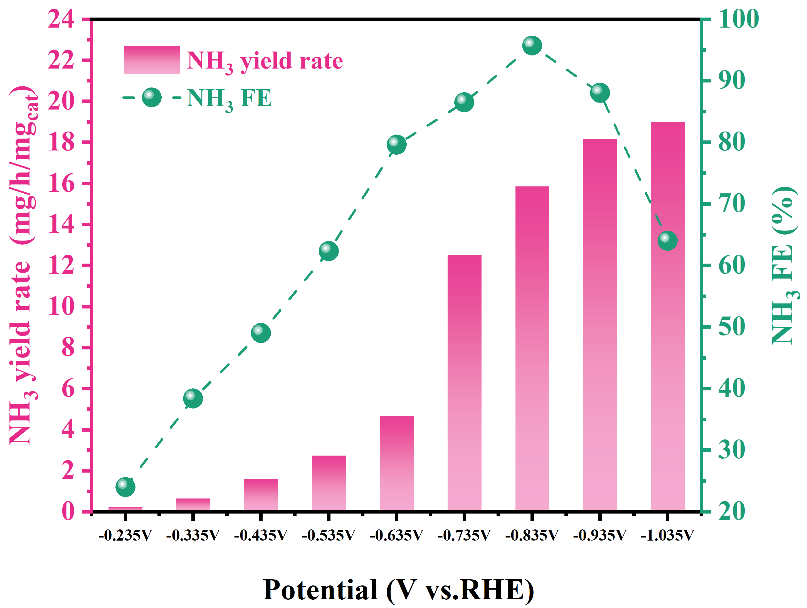


**Fig.S15 NH_3_ yields and NH_3_FEs of of Cu/N-SnS_2-x_ at different potentials.**

**Fig.S16 (a) LSV curves of Cu/N-SnS_2-x_ in 0.1M NaOH and 0.1M NaOH+ 0.1M NaNO_3_. (b) LSV curves of Cu/N-SnS_2-x_ in 0.1M NaOH , 0.1M NaOH+ 0.1M NaNO_3_ and 0.1M NaOH+ 0.1M NaNO_2_**

**Fig.S17 (a)CA curves of Cu/N-SnS_2-x_ at given potentials; (b) UV spectra of Cu/N-SnS_2-x_ at given potentials; (c) NH_3_ production rate of Cu/N-SnS_2-x_ at given potentials; (d) Faraday efficiency of Cu/N-SnS_2-x_ at given potentials**


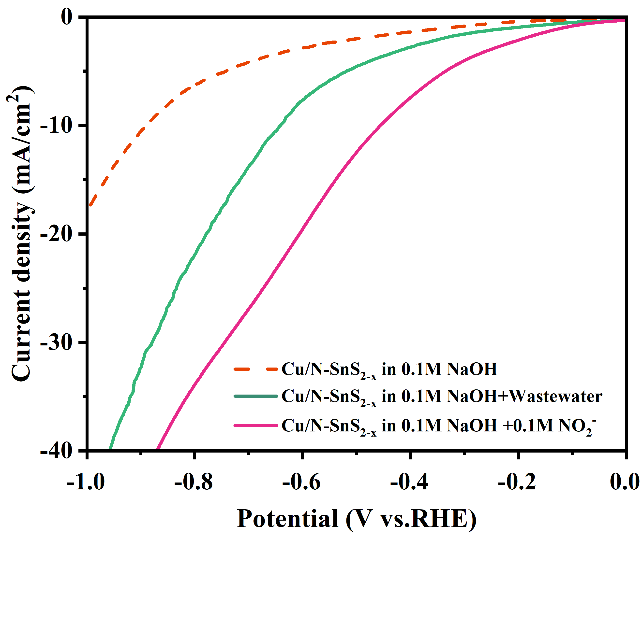


**Fig.S18 LSV curves of Cu/N-SnS_2-x_ in 0.1M NaOH , 0.1M NaOH+ 0.1M NaNO_2_ and 0.1M NaOH+ Wastewater.**

**Fig.S19 (a)CA curves of Cu/N-SnS_2-x_ at given potentials; (b) UV spectra of Cu/N-SnS_2-x_ at given potentials; (c) NH_3_ production rate of Cu/N-SnS_2-x_ at a given potentials; (d) Faraday efficiency of Cu/N-SnS_2-x_ at a given potentials**


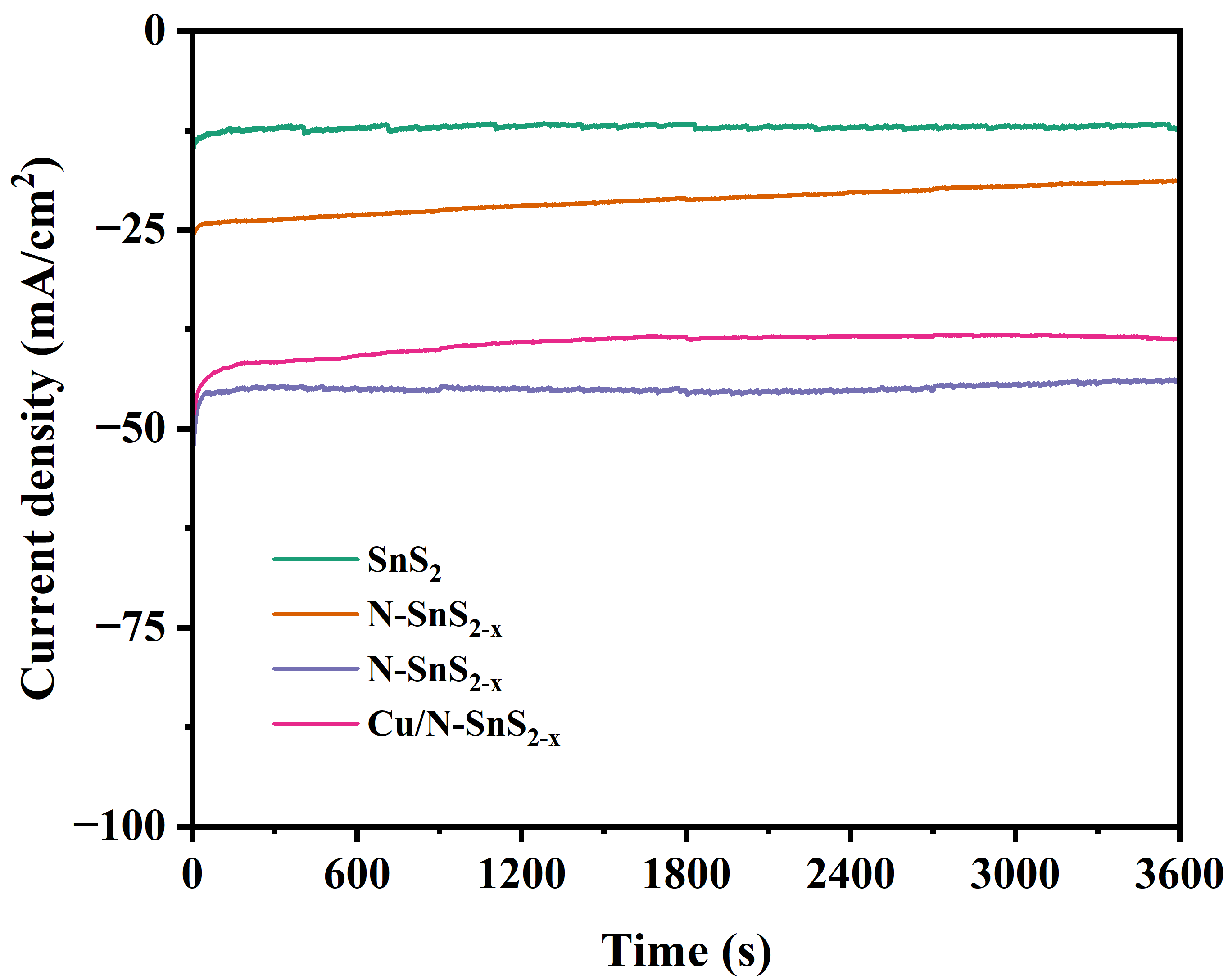


**Fig. S20. Chronoamperometry curves of different samples.**


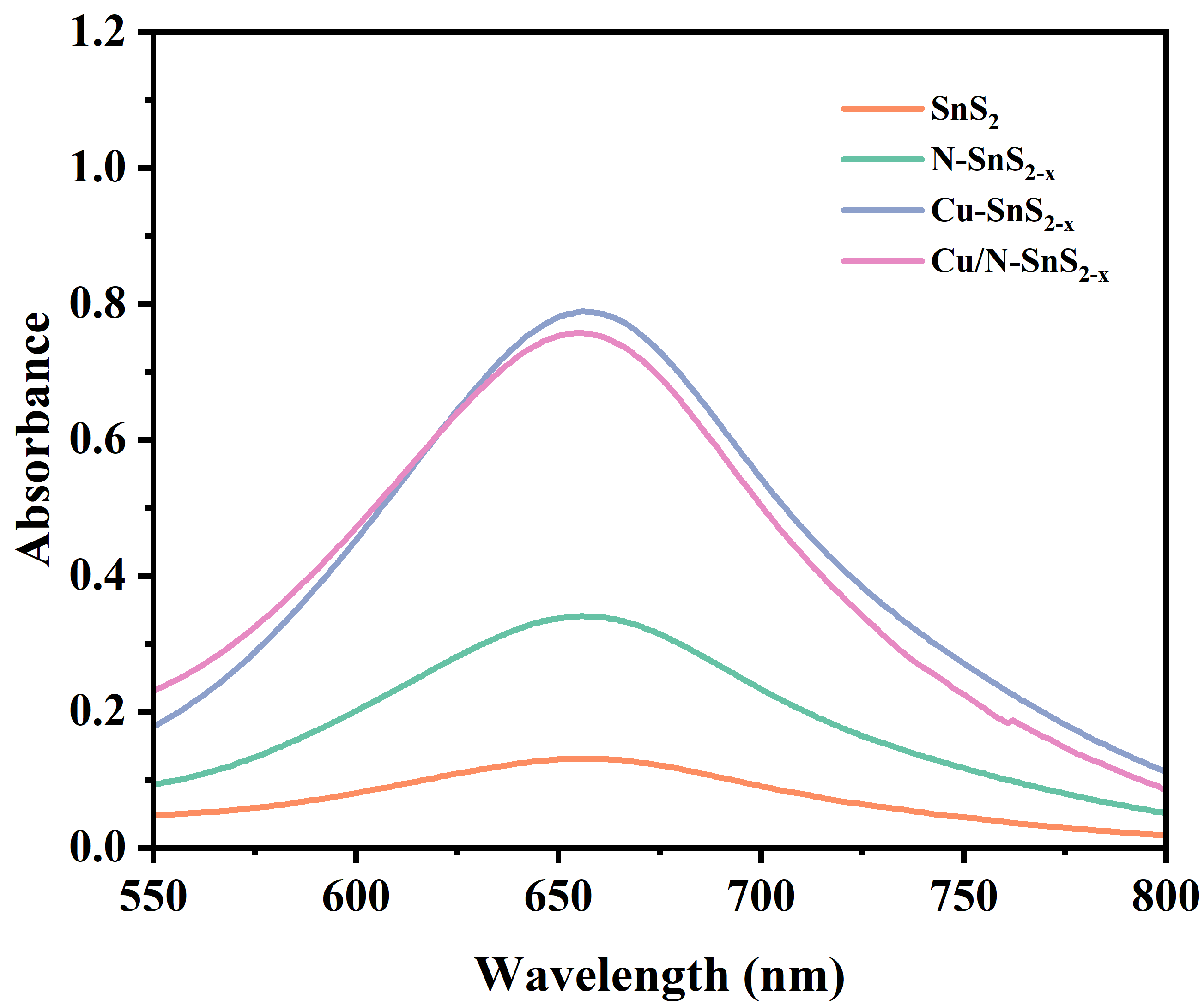


**Fig. S21. UV-vis spectra of different samples.**


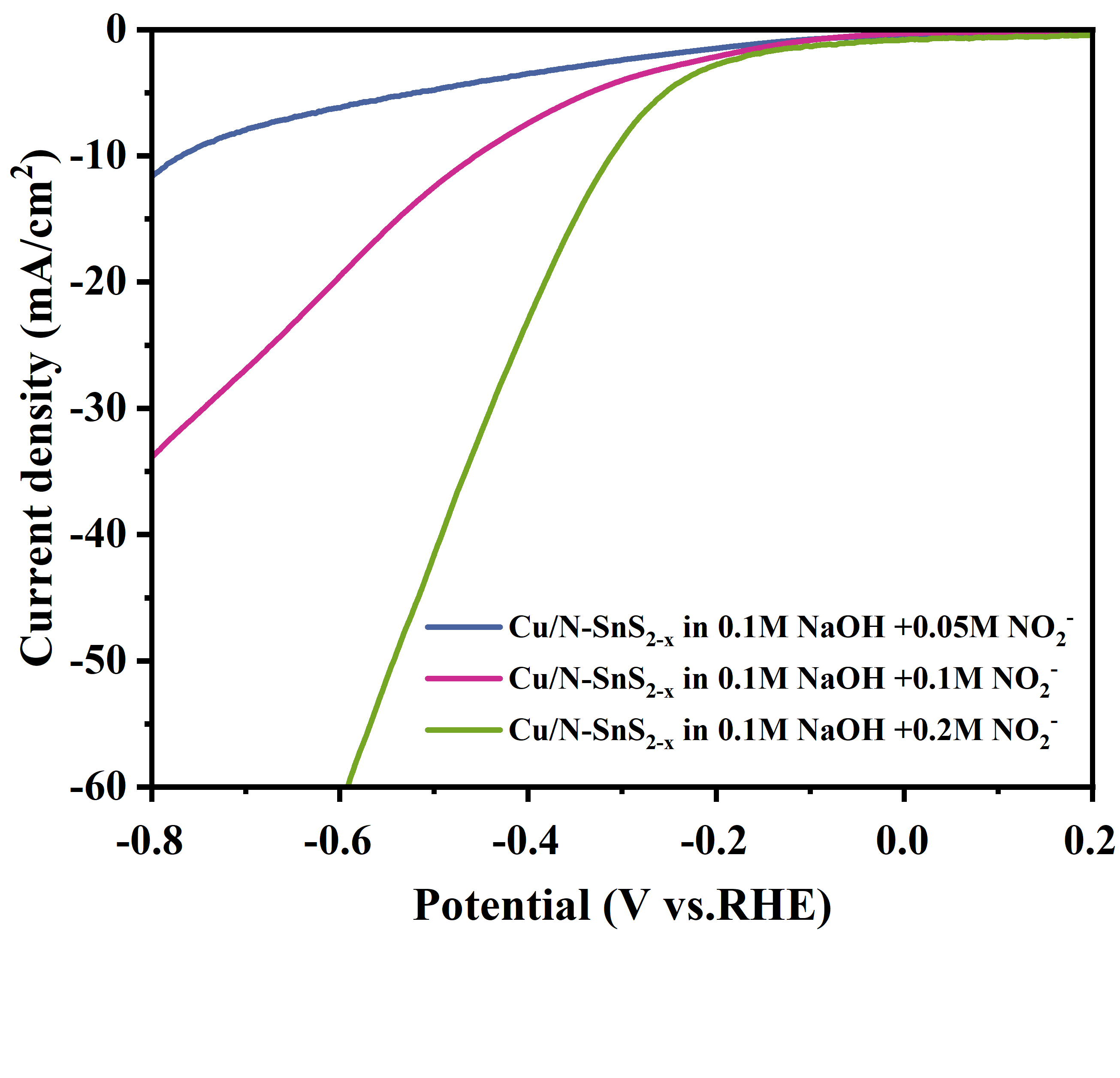


**Fig. S22. LSV curves of Cu/N-SnS_2-x_ under different NO_2_^-^ concentrations**

\


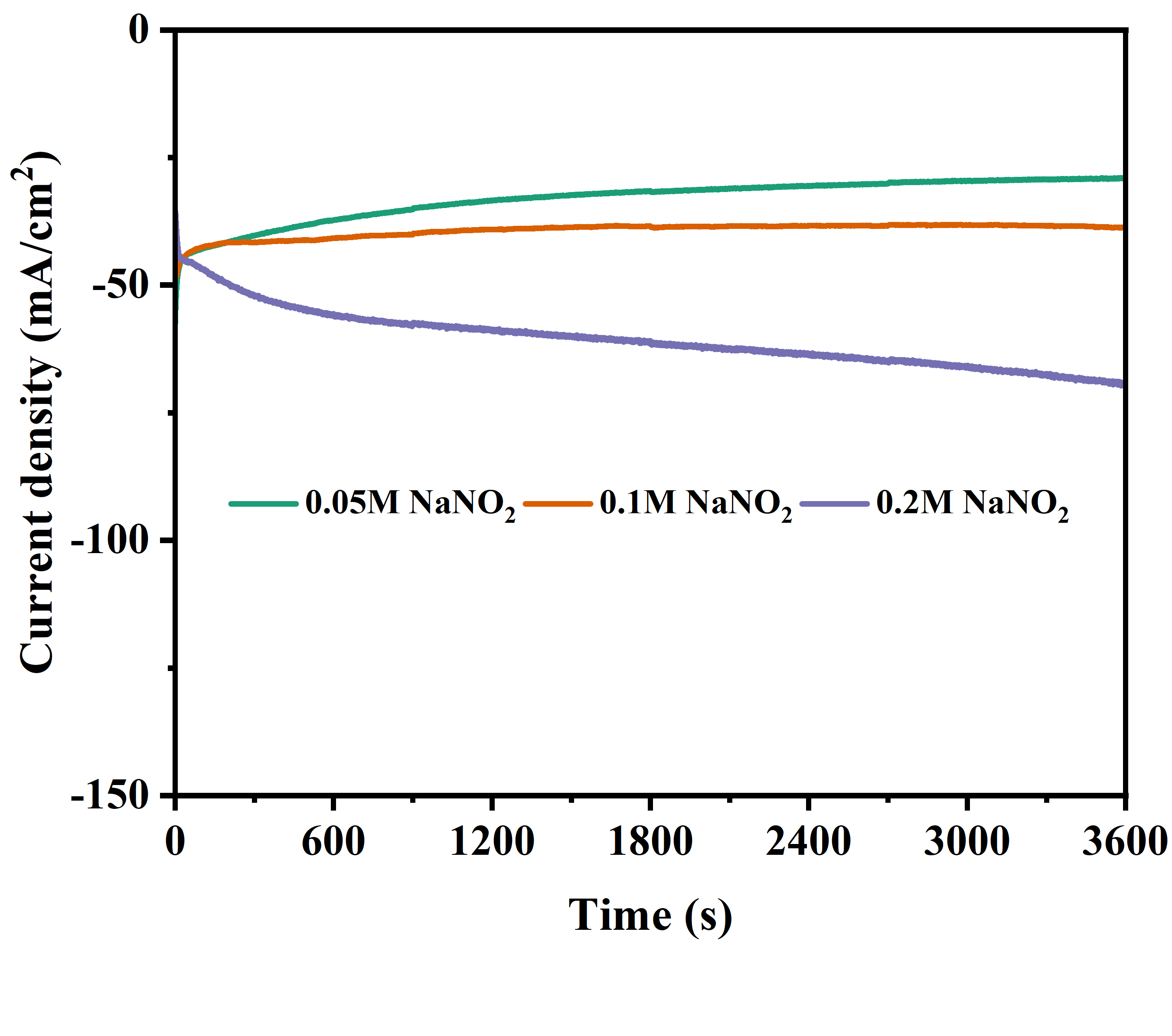


**Fig. S23. Chronoamperometry curves of Cu/N-SnS_2-x_ under different NO_2_^-^ concentrations**


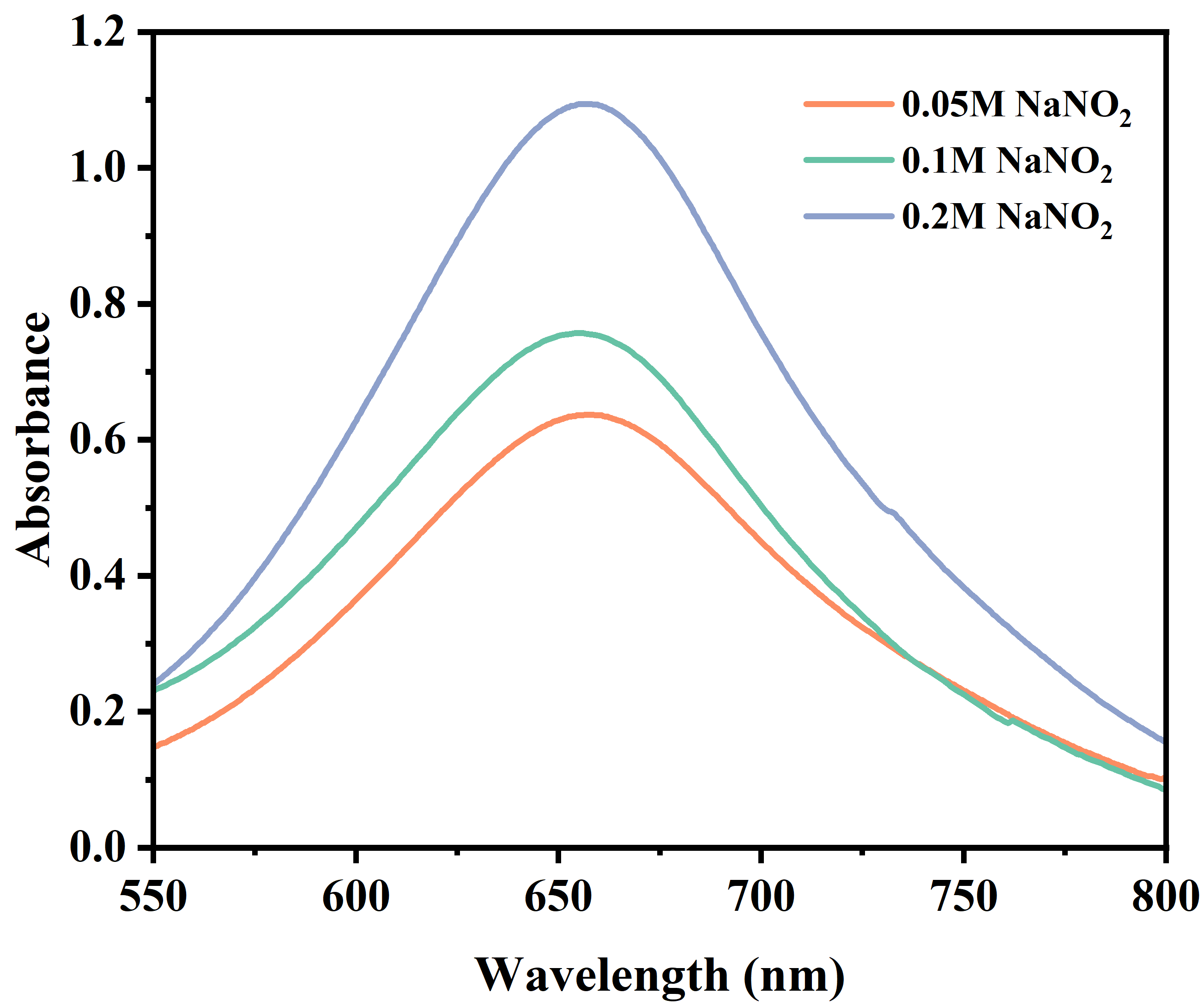


**Fig. S24. UV-vis spectra of Cu/N-SnS_2-x_ under different NO_2_^-^ concentrations.**

**Fig. S25. NO_2_RR test criterion schematic**

**
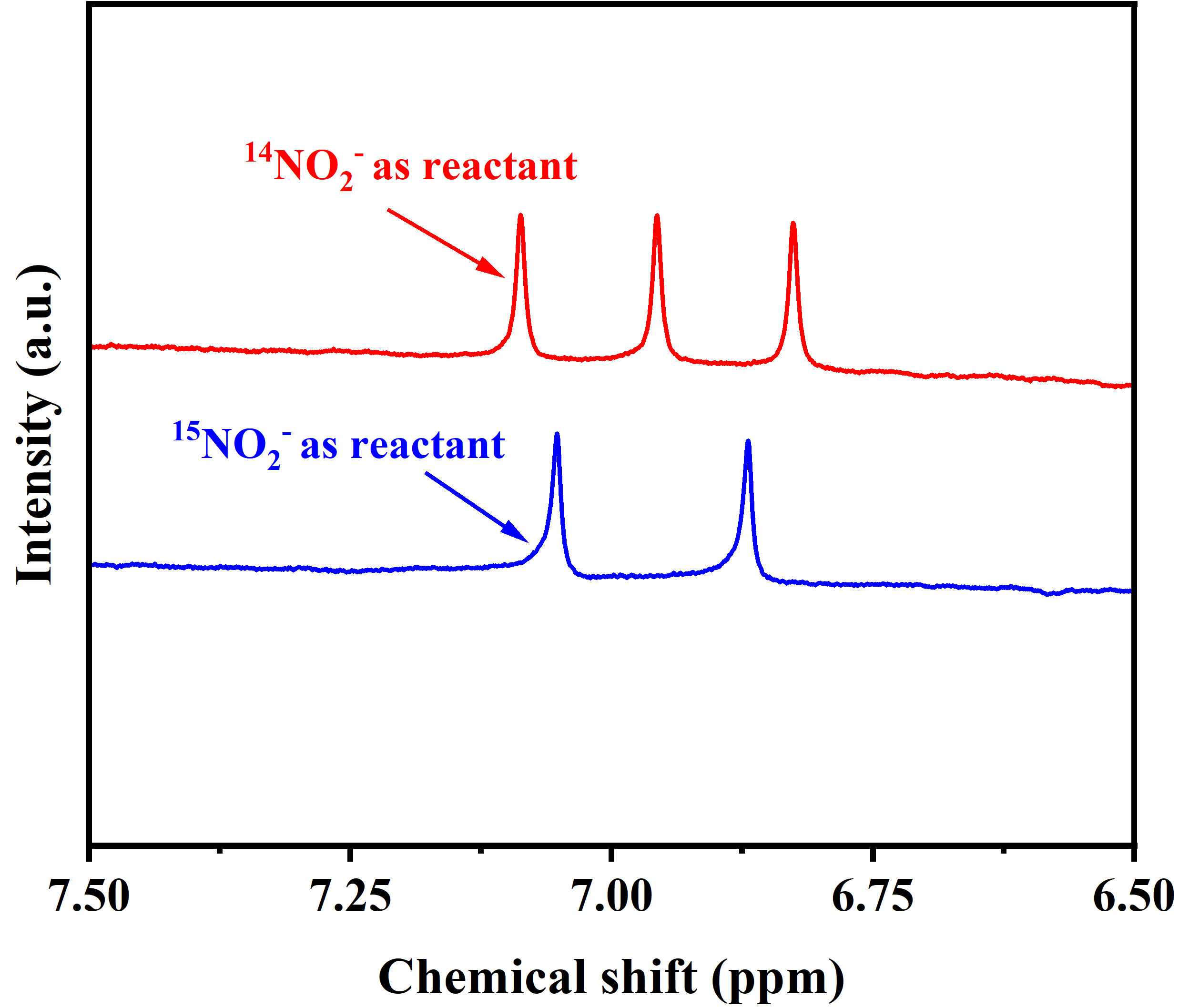
**

**Fig. S26. ^1^H-NMR spectra.**


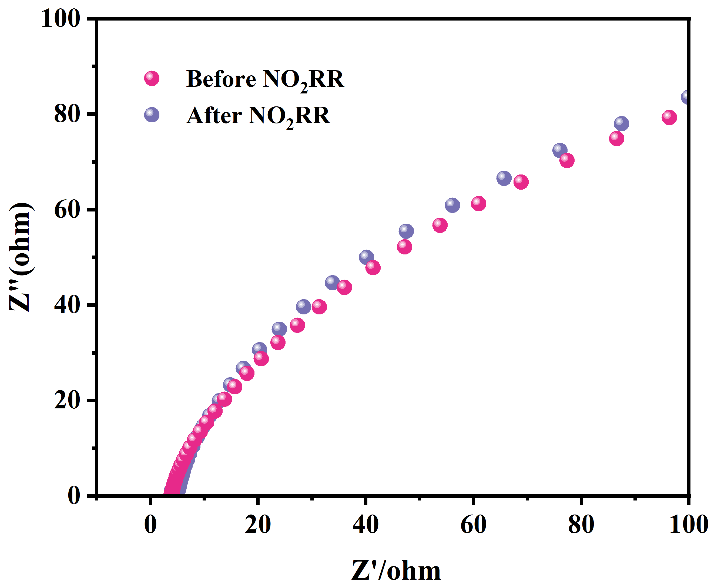


**Fig.S27 EIS results of Cu/N-SnS_2-x_ before and after NO_2_RR**

***
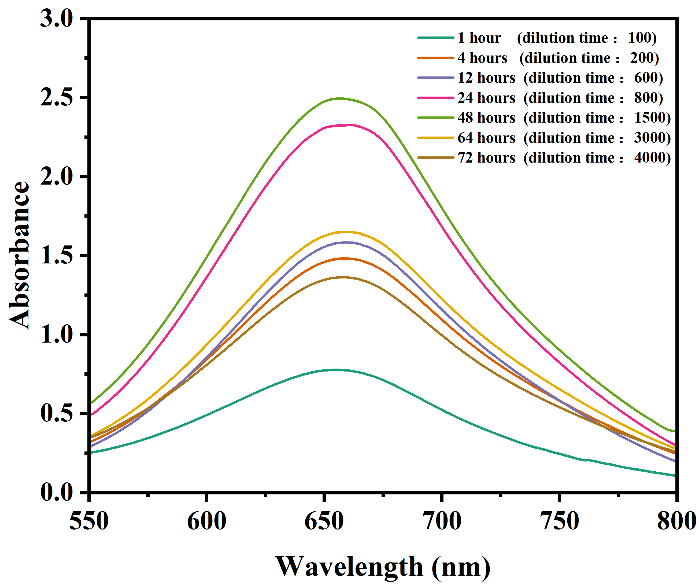
***

**Fig.S28 UV-vis spectras of Cu/N-SnS_2-x_ under different NO_2_RR time**

***
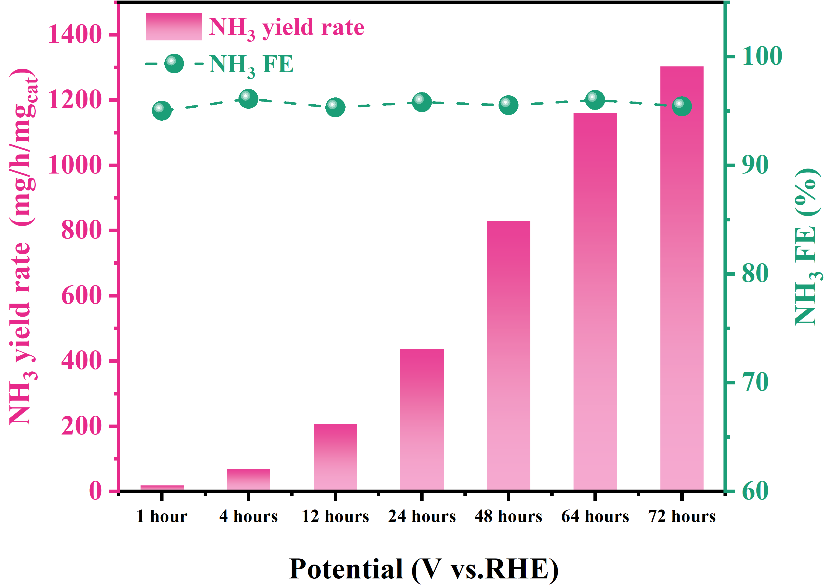
***

**Fig.S29** **NH_3_ yields and NH_3_FEs of of Cu/N-SnS_2-x_ under different NO_2_RR time.**

**Fig. S30.** **XRD patterns of Cu/N-SnS_2-x_ before and after Nitrite reduction reaction**

**Fig. S31. TEM image of Cu/N-SnS_2-x_ after Nitrite reduction reaction**

**Fig.S32** **HRTEM image of Cu/N-SnS_2-x_ after the long-term test.**

**Fig.S33(a) XPS Cu2p spectra (b) XPS N 1s spectra. (c) XPS Sn3d spectra. (d) XPS S2p spectra.**


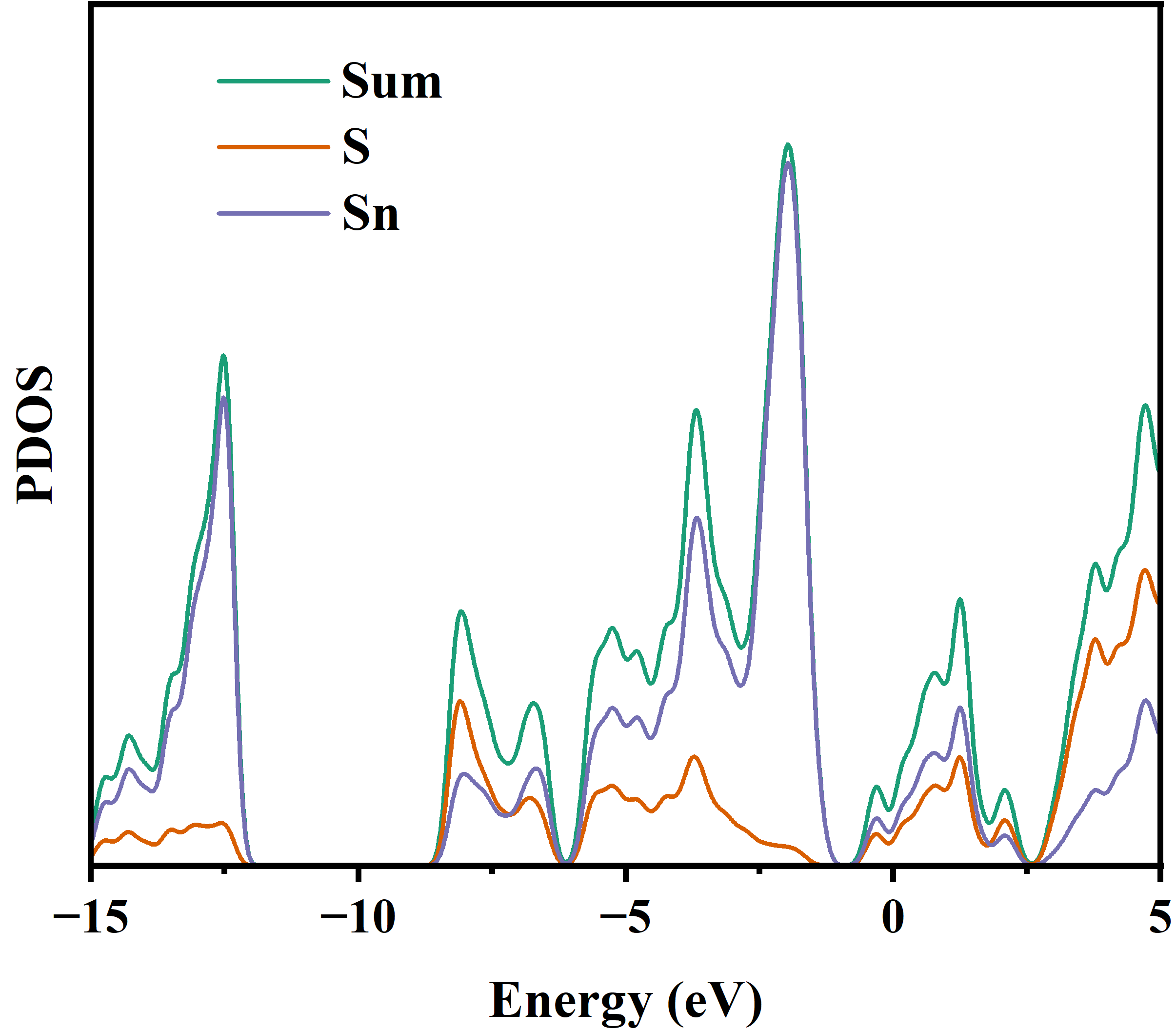


**Fig.S34 PDOS of SnS_2-x_**


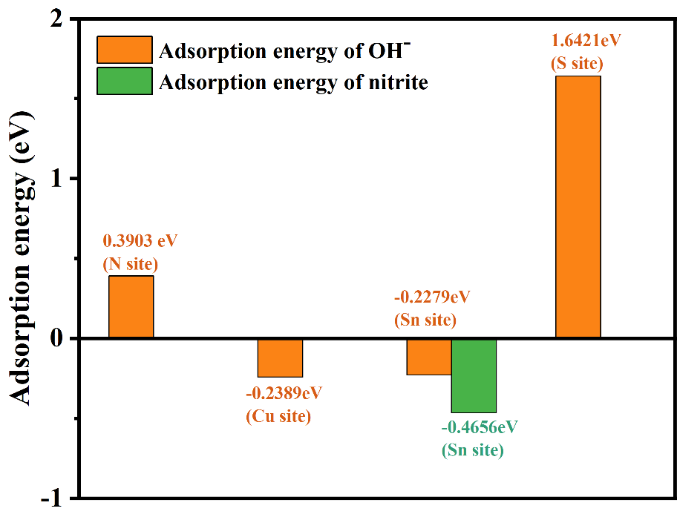


**Fig.S35** **Adsorption energy comparison of nitrite and OH- on different active sites of Cu/N-SnS_2-x_**

| **Elements** | **Wt.%** |
| --- | --- |
| Cu | 5.76% |

**Table.S1.** **ICP-OES results of Cu**

| **SnS_2_ (Spins)** | **Cu/N-SnS_2-x_ (Spins)** |
| --- | --- |
| **1.673*10^16^** | **7.038*10^17^** |

**Table S2 Comparison of the number of unpaired electrons of SnS_2_ and Cu/N-SnS_2-x_**

| **Catalyst** | **NH_3_ yield rate**  **(mg h^-1^ mg_cat_^-1^)** | **NH_3_ FE**  **(%)** | **Electrolyte** | | **Ref.** |
| --- | --- | --- | --- | --- | --- |
| **Cu/N-SnS_2-x_** | **18.15** | **95.7** | | **0.1 MNaOH+0.1 M NaNO_2_** | **This work** |
| **Co_3_O_4_/NiFe LDH** | **4.27** | **96.53** | | **0.1 MNaOH+0.1 M NaNO_2_** | **[1]** |
| **C-NiWO_4_/NF** | **10.97** | **97.6** | | **0.1 MNaOH+0.1 M NaNO_2_** | **[2]** |
| **Cu_3_P@TiO_2_** | **27.2** | **97.1** | | **0.1 MNaOH+0.1 M NaNO_2_** | **[3]** |
| **Ni@JBC-800** | **4.08** | **83.4** | | **0.1 MNaOH+0.1 M NaNO_2_** | **[4]** |
| **MoS_2_ NSs** | **8.99** | **93.52** | | **0.5 MNa_2_SO_4_+0.1 M NaNO_2_** | **[5]** |
| **Ni-NSA-V_Ni_** | **4.2** | **88.9** | | **0.2 MNa_2_SO_4_+200 ppm NaNO_2_** | **[6]** |
| **Ru–TiO_2_** | **26.52** | **98.9** | | **0.1 MNaOH+0.1 M NaNO_2_** | **[7]** |
| **NiMoO_4_** | **18** | **94.49** | | **0.5 MNa_2_SO_4_+0.1 M NaNO_2_** | **[8]** |
| **Ni_2_P nanosheet** | **18.7** | **90.2** | | **0.1 MPBS+200 ppm NaNO_2_** | **[9]** |
| **Co_1_/C_3_N_4_** | **18.36** | **97.9** | | **0.5 M Na_2_SO_4_ + 0.1 M NaNO_2_** | **[10]** |
| **L-Pd** | **8.68** | **95.2** | | **0.5 M Na_2_SO_4_ + 0.1 M NaNO_2_** | **[11]** |
| **u-Cu** | **8.4** | **94.7** | | **0.5 M Na_2_SO_4_ + 0.1 M NaNO_2_** | **[12]** |
| **ZnFe_2_O_4_** | **9.99** | **95.7** | | **0.1 M NaOH+0.1 M NaNO_2_** | **[13]** |

**Table S3.****Comparison with various electrocatalysts for NO_2_RR.**

| **Model** | **The reaction energy** | **Zero-Point energy** | **Entropy corrections** |
| --- | --- | --- | --- |
| **Cu/N-SnS_2-x_*NO_2_** | **-226.223 eV** | **0.243 eV** | **0.1274 eV** |
| **Cu/N-SnS_2-x_*NO** | **-219.447 eV** | **0.168 eV** | **0.0903 eV** |
| **Cu/N-SnS_2-x_*N** | **-214.072 eV** | **0.085 eV** | **0.0695 eV** |
| **Cu/N-SnS_2-x_*NH** | **-218.0215 eV** | **0.396 eV** | **0.3803 eV** |
| **Cu/N-SnS_2-x_*NH_2_** | **-222.3428 eV** | **0.731 eV** | **0.7116 eV** |
| **Cu/N-SnS_2-x_*NH_3_** | **-226.6895 eV** | **1.031 eV** | **0.9869 eV** |

**Table.S4: The reaction energy, zero-point energy, and entropy corrections of each intermediate on Cu/N-SnS_2-x_**

| **Model** | **The reaction energy** | **Zero-Point energy** | **Entropy corrections** |
| --- | --- | --- | --- |
| **Cu-SnS_2-x_*NO_2_** | **-224.767 eV** | **0.288 eV** | **0.12 eV** |
| **Cu-SnS_2-x_*NO** | **-218.041 eV** | **0.171 eV** | **0.09 eV** |
| **Cu-SnS_2-x_*N** | **-210.4475 eV** | **0.093 eV** | **0.073 eV** |
| **Cu-SnS_2-x_*NH** | **-216.197 eV** | **0.403 eV** | **0.38 eV** |
| **Cu-SnS_2-x_*NH_2_** | **-221.488 eV** | **0.743 eV** | **0.71 eV** |
| **Cu-SnS_2-x_*NH_3_** | **-225.275 eV** | **1.522 eV** | **0.48 eV** |

**Table.S5: The reaction energy, zero-point energy, and entropy corrections of each intermediate on Cu-SnS_2-x_**

References

[1] FENG Y, REN J-T, WANG H-Y, et al. Core–shell heterojunction engineering of Co_3_O_4_/NiFe LDH nanosheets as bifunctional electrocatalysts for efficient reduction of nitrite to ammonia [J]. Inorganic Chemistry Frontiers, 2023, 10(15): 4510-8.

[2] QIU H, CHEN Q, ZHANG J, et al. NiWO_4_ nanoparticles with oxygen vacancies: high-efficiency electrosynthesis of ammonia with selective reduction of nitrite [J]. Inorganic Chemistry Frontiers, 2023, 10(13): 3909-15.

[3] CAI Z, ZHAO D, FAN X, et al. Rational construction of heterostructured Cu_3_P@TiO_2_ nanoarray for high‐efficiency electrochemical nitrite reduction to ammonia [J]. Small, 2023, 19(30): 2300620.

[4] LI X, LI Z, ZHANG L, et al. Ni nanoparticle-decorated biomass carbon for efficient electrocatalytic nitrite reduction to ammonia [J]. Nanoscale, 2022, 14(36): 13073-7.

[5] YI L, SHAO P, LI H, et al. Scalable synthesis of MoS_2_ nanosheets electrocatalyst towards high-efficiency nitrite reduction to ammonia [J]. Journal of Power Sources, 2023, 559: 232668.

[6] WANG C, ZHOU W, SUN Z, et al. Integrated selective nitrite reduction to ammonia with tetrahydroisoquinoline semi-dehydrogenation over a vacancy-rich Ni bifunctional electrode [J]. Journal of Materials Chemistry A, 2021, 9(1): 239-43.

[7] REN Y, ZHOU Q, LI J, et al. Ruthenium doping: An effective strategy for boosting nitrite electroreduction to ammonia over titanium dioxide nanoribbon array [J]. Journal of Colloid and Interface Science, 2023, 645: 806-12.

[8] WANG G, CHEN Q, ZHANG J, et al. NiMoO_4_ nanorods with oxygen vacancies self-supported on Ni foam towards high-efficiency electrocatalytic conversion of nitrite to ammonia [J]. Journal of Colloid and Interface Science, 2023, 647: 73-80.

[9] WEN G, LIANG J, ZHANG L, et al. Ni_2_P nanosheet array for high-efficiency electrohydrogenation of nitrite to ammonia at ambient conditions [J]. Journal of Colloid and Interface Science, 2022, 606: 1055-63.

[10] ZHAO H, XIANG J, SUN Z, et al. Electroreduction of nitrite to ammonia over a cobalt single-atom catalyst [J]. ACS Sustainable Chemistry & Engineering, 2024, 12(7): 2783-9.

[11] QU W, WU T, WANG J, et al. Low-coordinated Pd metallene promotes the electrochemical reduction of nitrite to ammonia [J]. New Journal of Chemistry, 2024, 48(10): 4346-50.

[12] ZHANG R, SHANG S, WANG F, et al. Electrocatalytic reduction of nitrite to ammonia on undercoordinated Cu [J]. Dalton Transactions, 2024, 53(8): 3470-5.

[13] XU C, LIANG Y, HE X, et al. ZnFe_2_O_4_ nanosheet array: a highly efficient electrocatalyst for ambient ammonia production via nitrite reduction [J]. Catalysis Science & Technology, 2024, 14(1): 57-61.
